# Improvements of particle filter optimization algorithm for robust optimization under different types of uncertainties^☆^

**DOI:** 10.1016/j.heliyon.2024.e41573

**Published:** 2025-01-03

**Authors:** Éva Kenyeres, Alex Kummer, János Abonyi

**Affiliations:** HUN-REN-PE Complex Systems Monitoring Research Group, Department of Process Engineering, University of Pannonia, Egyetem u. 10, P.O. BOX 158, Veszprém, H-8200, Hungary

**Keywords:** Robust optimization, Uncertainty quantification, Stochastic objective function, Sampling-based techniques

## Abstract

This paper introduces a methodology for handling different types of uncertainties during robust optimization. In real-world industrial optimization problems, many types of uncertainties emerge, e.g., inaccurate setting of control variables, and the parameters of the system model are usually not known precisely. For these reasons, the global optimum considering the nominal values of the parameters may not give the best performance in practice. This paper presents a widely usable sampling-based methodology by improving the Particle Filter Optimization (PFO) algorithm. Case studies on benchmark functions and even on a practical example of a styrene reactor are introduced to verify the applicability of the proposed method on finding robust optimum, and show how the users can tune this algorithm according to their requirement. The results verify that the proposed method is able to find robust optimums efficiently under parameter and decision variable uncertainties, as well.

## 1 Introduction

Looking for optimums with high objective value is not a satisfactory strategy nowadays in our rapidly changing world. In an engineering system, many uncertain factors are present and affect system performance. Therefore, stability is also a crucial aspect to be taken into account. This paper offers a solution to this challenging issue by providing a methodology for finding robust optimums in the case of different types of uncertainties. The set of expected trade-off level between peak performance and stability is integrated into the methodology besides proposing an extensive analysis on how the tunable elements affect which optimum is found. The proposed robust optimization technique can be used flexibly even in the presence of multiple types of uncertainties adapted to the requirements of users.

Optimization techniques are widely used in practical problems where finding the best set of values for some variables is desired in order to reach the best solution. The aforementioned variables are denoted as decision variables in the optimization terminology, that are members of the so-called objective function which describes the optimization goal. The goal of the optimization can be, e.g., creating an accurate model; improving the efficiency of an equipment; making the scheduling more efficient; or maximizing the profit of a technology by reducing the cost or increasing the benefit that can be gained from a given amount of resources [1]. The aims of Industry 5.0 (e.g., sustainability, resilience) bring new aspects into consideration, as well [2]. Resilience, for example, is a crucial factor when optimal operating conditions of a complex system need to be determined [3]. Political and social pressures are also significant to increase energy efficiency and reduce pollution while making processes more robust and reliable by operating them stable [4].

In most cases, the global optimum of the objective function is searched for; however, if uncertainties are present in the process system or its model, it may not give the best solution. In an optimization problem from the industrial field, many uncertain factors can be identified according to their sources [5]: on the one hand, randomness of the external circumstances, like unpredictability of demand or prices, has to be taken into account; on the other hand, limited knowledge about the technology causes the inaccuracy of the system model or its parameters were estimated based on imprecise measurement data; process inherent uncertainties during the operation, e.g., variations in flowrate or temperature fluctuations also have to be considered (internal uncertainties [6]). These uncertainties all affect the optimization problem; therefore, a less sensitive local optimum may perform better in practice than the global one. The stability (sensitivity) of the optimum is referred to as its robustness. To overcome the problem of considering the stability of the optimum besides its performance during the search, and to handle uncertainties that make it more difficult to find the solution that reaches the best performance in practice, robust optimization techniques were developed [7].

Optimization under uncertainties can be classified into “here and now” and “wait and see” problems [8]. The latter means that the deterministic problem is solved after an observation is made on the uncertain elements. A robust optimization technique called Γ-optimality approach was developed based on this idea used to solve the shortest path problems in [9]. The two-stage and multistage optimization also integrate the “wait and see” principle [10]. However, in most cases, it is not possible to wait for the observations or gain more information about the uncertain elements during decision making. These are called “here and now” problems, where uncertainties are handled by probabilistic measures. Thereby, uncertainty quantification (UQ) is an essential part of robust optimization techniques [11].

UQ methods can be classified as intrusive and nonitrusive techniques [12]. Intrusive methods formulate a surrogate model by the modification of the original one, e.g. stochastic metamodel methods or polynomial chaos expansion (PCE), which investigate the effect of uncertain variables on the objective function value. Bayesian Optimization technique has also been used to create the probabilistic surrogate model of an unknown objective function [13]. These intrusive methods are widely used to avoid solving computationally intensive models, e.g. CFD simulation multiple times [14], [15]. On the other hand, nonintrusive techniques are more convenient for complex systems, especially when many types of uncertainties are present, as they only require multiple calculations of the original model. Thereby, it can be avoided to generate a surrogate model, which is often a difficult task in the case of systems with high complexity. The user only has to deal with somewhat higher computational demand [12].

In the case of nonintrusive methods, scenario-based techniques using uncertainty sets are very popular nowadays to handle uncertainties [10]. Two-stage and multistage stochastic programming optimize over the expected outcome of scenarios in multiply stages, taking advantage of some uncertainties become certain for the later stages of the optimization by making decisions in the early stages. The technique was described through a process network problem in [10]. The robust counterpart methodology of Ben-tal and Nemirovski also creates uncertainty sets to represent uncertain factors (e.g., convex sets considering all possible values or ellipsodial ones by eliminating rare events) [16]. The worst case approach takes into account the worst case condition of the uncertainties, thus ensuring the robustness of the solution, which was used, e.g., to minimize the total cost of energy hubs under fluctuations in prices [17]. However, in these cases, the problem can be overdesigned. It means that, despite the fact that the risk caused by uncertainties has been eliminated, we might fail getting the most out of the technology and performance losses are expected in practice [7].

The above-mentioned intrusive techniques eliminate uncertainties before solving the optimization problem by transforming the models to a deterministic form. On the other hand, the nonintrusive UQ methods for robust optimization to be able to represent the known probability distribution or uncertainty set of the uncertain variables and calculate the objective value for a certain point of the search space, that is computationally expensive. In this paper, the uncertainties are handled in parallel with the optimization problem and are aggregated only at the end of it. With this strategy, uncertainties can be flexibly handled, and even their combined effect can be considered. For this purpose, sampling-based techniques are offered to sample from the probability distribution of the uncertain variables, and propagate the sample elements through the system model that is being optimized. In contrast to the above-mentioned nonintrusive techniques, the exploration regarding uncertain variables and on the search space are performed simultaneously in our proposed method, which is a major difference, e.g., from the robust counterpart method, and can reduce computational demand. The solution gained by these techniques may carry some risk, but the expected revenue will be better with high probability. Thereby, as far as uncertainties allow, a practically optimal solution can be found [18].

Nowadays, population-based algorithms are also quite popular in the field of optimization. The increased computational capacity allows to propagate multiple candidate solutions on the search space simultaneously, and store their data [19]. These algorithms are often inspired from social behavior of animals, e.g., when a hunting population chases a prey (Grey Wolf Optimizer [20]), or a group of birds looking for food whose location is unknown (Particle Swarm Optimization [21]). In this kind of algorithms, the displacement vector of the individuals, i.e., candidate solutions is determined in every iteration to get closer to the chased object whose assumed location is updated continuously based on the population. However, to integrate sampling-based uncertainty handling into the optimization, an algorithm with probabilistic approach seems more preferable, such as the recently released Particle Filter Optimization (PFO) algorithm [22]. In this case, not the displacement and route of the candidate solutions (called particles here) is estimated, but they are rearranged in each iteration according to some probabilistic rules.

Most of the case studies in the available literature consider only one type of uncertainty when searching for the robust optimum of the system. The optimal operation scheme of a hydropower station is estimated under process input uncertainty, namely, the inaccuracy of streamflow forecast in [23]. Parameter uncertainties are considered during the optimization of the operation of a grinding circuit in [6]. Operational uncertainty such as temperature variation was handled by a PCE-based technique in case of a so-called power-to-ammonia system that aims to store wind energy in ammonia serving as an energy carrier in [15]. However, no work was found that suggests a common method for handling multiple types of uncertainties.

Thereby, the present paper set out to provide a methodology based on Particle Filter Optimization (PFO) algorithm, which allows to manage different types of uncertainties together such as input, operational, model, and objective parameter uncertainty. PFO is a population-based optimization algorithm that was first introduced by Zhou as an analogue of the particle filtering state estimation framework [22]. Although the algorithm is quite novel and rarely documented, an extensive comparative analysis regarding the performance of finding the global optimum and some improvements were also suggested recently [24], that gives the basis for this work, as well. The probabilistic nature of the algorithm makes it promising regarding integrating uncertainties in the form of probability distributions [25].

The novelty of the paper is a sampling-based technique using the PFO algorithm for handling different types of uncertainties during optimization, aiming to find a robust optimum that is stable even under inaccurately known parameters and/or decision variables with fluctuating nature. The PFO algorithm is improved by integrating uncertainty handling into it. There is no need for the creation of a surrogate model, the original system model and objective function is used. Uncertainties are not aggregated before the optimization, but are implemented into the search strategy. Therefore, the proposed method is flexible regarding the number and types of uncertain factors, i.e., uncertainties modeled by any kind of probability distribution can be considered easily, and even multiple uncertain factors at the same time. The desired level of performance-robustness trade-off can be easily interpreted into the algorithm, controlling the search strategy by the tunable elements of the algorithm according to the requirements of the user. The proposed method is tested on different benchmark functions, and an example from the chemical engineering field is also introduced, where safety and reliability goals require operating the technology at a robust optimum.

The contributions of the paper are the following:

• Sampling-based improvements of the general PFO algorithm were proposed to make it applicable for robust optimization under parameter or decision variable uncertainty.
• The PFO and the widely used PSO algorithms were compared in finding the most robust ones from multiple global optimums.
• A tuning method for the proposed PFO-X algorithm was provided considering user preferences regarding the performance-robustness trade-off, which was tested on benchmark functions with multiple optimums.
• The case study of a styrene reactor was introduced to illustrate the importance of robust solutions regarding the system parameters and test the proposed PFO-P algorithm for finding these.

The roadmap of the paper is the following. Section 2 contains the methodological details about the robust optimization by PFO algorithm: Section 2.1 formalizes the general robust optimization problem and the effect of parameter and decision variable uncertainties on the objective value; Section 2.2 introduces the general PFO algorithm in detail (Section 2.2.1), together with its improvements in considering decision variable and parameter robustness during the search (Section 2.2.3-2.2.4) after giving an insight into the robustness aspects of a process system (Section 2.2.2). Section 3 summarizes the results of testing the proposed techniques on various benchmark functions regarding decision variable robustness (Section 3.1), and a practical case study of a styrene reactor regarding parameter robustness (Section 3.2).

## 2 Robust optimization with particle filter optimization algorithm

As the paper aims to integrate different types of uncertainties into the robust optimization problem, a methodology based on sampling is provided in this section, which enables sampling the space of the uncertain parameters and the decision variables during the optimization. The probabilistic nature of the Particle Filter Optimization (PFO) algorithm makes it easy to handle uncertainties simultaneously with the optimum search, and finally converging to a robust optimum that is stable regarding the uncertain parameters and/or fluctuating decision variables, as well.

### 2.1 Formalization of an optimization problem under uncertainty

In a single-objective optimization problem, the task is finding the optimal values of the decision variables ($x^{⁎}$) to reach the pre-defined objective which can be profit maximization or consumption minimization, for example. The objective value (*z*) can be calculated by the objective function (H(⋅)) with parameters **p**, that describes its dependence on the decision variables as:

z=H(x,p)

In engineering problems, the decision variables usually represent the system inputs or operational parameters that can be influenced. In this case, the objective function can depend directly on the system outputs (**y**) and the system inputs (**x**), as well. The connection between **x** and **y** is described by the system model (g(⋅)) with Θ parameters.

An unconstrained optimization problem in this case can be formalized as:

(1) $$x_{global}^{⁎}=\max_{x}H(x,y,p)$$

(2) subject toy=g(x,Θ)

(3) $$\;\;x^{L}\leq x\leq x^{U}$$

where H(⋅) refers to the objective function as a maximisation problem, **p** denotes to its parameters, **x** to the decision variables that covers the operational variables of the process system, and **y** to the outputs of the system model. Function g(⋅) in Equation (2) represents the implicit form of the system model with **Θ** parameters, which serves as an optimization constraint in the present formalization. In Equation (3), the lower ($x^{L}$) and upper bounds ($x^{U}$) of the decision variables are fixed.^1^

Three main types of uncertainties are considered during the optimization: uncertain settings of the operational variables ($U_{x}$), parameters of the system model ($U_{\Theta }$) and that of the objective function ($U_{p}$). These uncertainties are represented by the random variables $\xi_{x}$, $\xi_{\Theta }$ and $\xi_{p}$, respectively, taken from predefined probability densities. For simplicity, these variables are defined as perturbance variables on **x**, **Θ** and **p**. Thereby, the expected values of all of their multivariate probability densities are zero and their covariance matrices have non-zero elements only in the main diagonal if their elements are independent from each other. (In some cases, dependencies between uncertain factors can be identified. For example, if the objective function describes the profit of a technology, **p** contains prices of raw materials and products, which can correlate with each other. This effect can be easily integrated to the methodology; however, in this paper, we consider uncertainties as independent factors for simplicity.) Having an *a priori* guess about their probability distribution, e.g., based on the nature of the uncertainty, makes it possible to integrate that knowledge into the proposed robust optimization framework, as will be seen in Section 2.2.3 and 2.2.4.

Considering these issues, Equation (1)-(3) can be reformalized as:

$$x_{robust}^{⁎}=\max_{x}H(x+\xi_{x},y,p+\xi_{p})\text{subject to}\;\;\;y=g(x+\xi_{x},\Theta +\xi_{\Theta })\;\;x^{L}\leq x+\xi_{x}\leq x^{U}$$

The effect of previously defined uncertainties on the objective value (*z*) can be determined by the *law of propagation of uncertainty* explicitly, that evaluates the uncertainty of *z* (marked by u(z)) based on the uncertainties of stochastic input variables and model parameters [26]:

(4) $$u^{2}(z)=\sum_{i=1}^{N_{x}}{\left(\frac{\partial f}{\partial x_{i}}\right)}^{2}u^{2}(x_{i})+\sum_{j=1}^{N_{\Theta }}{\left(\frac{\partial f}{\partial \Theta_{j}}\right)}^{2}u^{2}(\Theta_{j})+\sum_{k=1}^{N_{p}}{\left(\frac{\partial f}{\partial p_{k}}\right)}^{2}u^{2}(p_{k})$$

where $x_{i}$, $\Theta_{j}$ and $p_{k}$ represents the elements of vectors **x**, **Θ** and **p**, respectively. $N_{x}$, $N_{\Theta }$ and $N_{p}$ refer to the number of decision variables, model parameters and objective function parameters, respectively. The uncertainties of the variables are denoted by u(⋅).

Equation (4) relates to the cases where *z* is linearly dependent on the uncertain variables. In this case, the square of the standard uncertainties of the decision variables and parameters weighted by sensitivity coefficients in the form of partial derivatives are summarized to estimate the uncertainty of objective value. Standard uncertainties can be represented by the standard deviation of the related variable based on *a priori* knowledge or measurement data. The sensitivity coefficients aim to describe how the change of a decision variable or parameter affects *z* [26].

However, in most practical cases, the connection between the objective value and the decision variables is nonlinear. This nonlinear feature appears in most system models and sometimes in the objective functions as well. In this case, the sensitivity coefficients have to be extended with higher-order terms in the Taylor series, that includes mixed derivatives. Evaluating these complex and high-order partial derivatives analytically requires a large effort. On the other hand, Equation (4) investigates the environment of one certain point of the search space. From an optimization point of view, it is unfavorable as the values of sensitivity coefficients differ and should be evaluated for every point of the search space. To avoid these problems, a method based on Monte Carlo (MC) simulation is suggested, in which a sample set is drawn from the probability density function (PDF) of stochastic input variables and parameters, and the model is evaluated to get the output, i.e., the system model and objective function to obtain *z* here, for every drawn element, thus avoiding the evaluation of sensitivity coefficients. This method can be easily used to investigate the environment of different points of the search space, i.e., different operating points in case of an industrial system, which can also be compared. (Of course, the computational demand depends on the complexity of the objective function and the uncertainty space.) Moreover, there is no restriction on the distribution of the uncertain variables, MC technique can flexibly handle any type of PDFs [18].

Particle Filter Optimization (PFO) is a sampling-based optimization algorithm, whose structure based on probabilistic principles carries the potential to integrate with the MC-method, thus handling uncertainty and applying for robust optimization problems as will be introduced in Section 2.2.

### 2.2 Particle filter optimization (PFO) for searching robust optimum

Particle Filter Optimization (PFO) technique is a novel population-based optimization algorithm that was developed based on the particle filtering (PF) state estimation framework. PFO has been mainly used for finding the global optimum of a function, however, its probabilistic background makes it possible to consider uncertainties during the optimization. In this section, the general PF framework and the PFO concept based on it are introduced briefly at first. Then, our improvements regarding handling decision variable and parameter uncertainties are presented, together with an outlook on how performance-robustness trade-off affects the search and which algorithm parameters can influence it.

#### 2.2.1 Introduction to the PF framework for optimization

**Figure 1:**
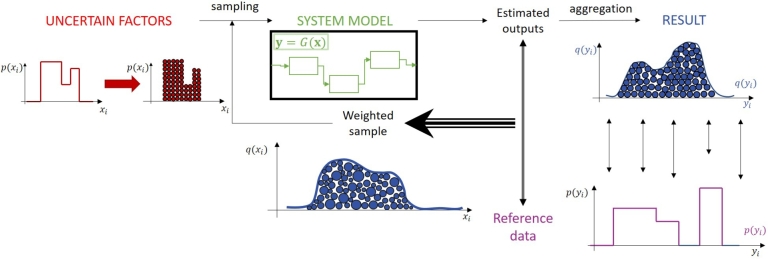
General scheme of particle filter-based algorithms. In this iterative Monte Carlo simulation, the elements of the resulted output sample are weighted according to a related reference data (e.g., noisy measurement data, uncertain expert knowledge or value of the objective function), thus forming a feedback by defining new distribution of the uncertain factors. These can be sampled again (resampling), continuing the iterative calculations until convergence.

Particle filter algorithms are sampling-based techniques with probabilistic background originally applied for state estimation [27]. The core of them is an MC simulation executed iteratively using importance sampling to weight the particles as can be seen in Fig. 1 [28].

During an MC simulation, the uncertain factors have to be represented by probability distributions at first. Samples called particles in the particle filtering field are drawn from these distributions, and the system outputs are computed using the system model. The obtained values can be aggregated into a probability distribution, whose expected value and confidence intervals can be determined from the sample set [18].

In the particle filtering scheme, the estimated system outputs are not aggregated immediately to get the result distributions, but they are compared to reference data that is often also available in the form of a probability distribution. It can be, e.g., noisy measurement data or expert knowledge in case of state or parameter estimation, for example [29], [30]. Thereby, the particles can be ordered or prioritized by weighting according to their similarity to the reference data, and thus the probability distributions of the output and input variables change. These are resampled on the one hand, to move the particles toward the most favorable places on the state space, and on the other hand, to get equally weighted particles again and avoid the degeneracy problem [27].

PF framework can be used for optimization as well. The basic Particle Filter Optimization (PFO) concept was proposed by Zhou first recently, as a unifying perspective on some other randomized optimization approaches [31]. In this case, the particles represent the candidate solutions that are moved on the search space, and the weights are generated based on their objective values that serve as reference data according to the scheme shown in Fig. 1. The asymptotical convergence of the search algorithm on the global optimum was also proven in a later work [32]. It was shown that for an observation sequence containing more and more information, the estimate approximates more and more global optimum (in case of the PFO) or true state (in case of PF). The ‘observation’ in case of the PFO is a monotonically increasing $y_{k}$ level which sets the boundary of zero-weight and positive-weight particles according to their objective value.

The PFO concept consists of three main parts: particle move, weight updating, and resampling related to the prediction, correction, and resampling steps of the PF framework, respectively.

1. *Particle move:* Every iteration begins with this step. At the end of the previous iteration, they accumulate in the high-performance points, and their position needs to be perturbed to explore the neighborhood of these points as well, and to distribute them better around that region. This concept is realized by adding a so-called transition noise ($u_{k}$) on the position of the particles, as described in Equation (5).
  (5) $$x_{k}^{i}={\overset{˜}{x}}_{k-1}^{i}+u_{k}^{i},$$
  where $x_{k}^{i}$ and ${\overset{˜}{x}}_{k-1}^{i}$ vectors denote the position of the *i*th particle on the (multidimensional) search space after the particle move step and before it corresponding to the position inherited from the previous iteration gained after the resampling step. $u_{k}^{i}$ refers to the transition noise vector, which is drawn from a zero-mean multivariate normal distribution with decreasing variance along the iterations for each particle. This element of the algorithm ensures the exploration of the search space in the earlier iterations and responsible for the convergence in the later ones. More information about the optimal trajectory of decrease, and a detailed analysis can be found in our previous work in [24].
2. *Weight updating:* After having the new position of the particles in iteration *k* ($x_{k}$), the corresponding objective values ($H(x_{k}$)) can be computed and weighting performed. In this case, the weighting strategy is the following: a so-called observation denoted by $y_{k}$ needs to be created as:
  (6) $$y_{k}=H_{max,k}-v_{k}$$
  where $v_{k}$ is a random number between the lowest ($H_{min,k}$) and highest ($H_{max,k}$) objective values of the particle set in the *k*th iteration.
  The created $y_{k}$ value serves as a boundary here, representing a level of the objective function and sorting the particles into two groups accordingly: the particles with worse performance (lower objective value) than $y_{k}$ get zero weights and will not be propagated to the next iteration, while particles with performance above the boundary level $y_{k}$ get positive weights ($w_{k}^{i}$) proportional to their performance controlled by the $\varphi_{k}(\cdot )$ probability density as:
  (7) $$w_{k}^{i}=\frac{\varphi_{k}(H(x_{k}^{i})-y_{k})w_{k-1}^{i}}{\sum_{i=1}^{N_{s}}\varphi_{k}(H(x_{k}^{i})-y_{k})w_{k-1}^{i}}$$
  where $N_{s}$ refers to the number of the particles.
  The steepness of $\varphi_{k}(\cdot )$ is user defined: the higher it is, the more nonzero weight particles are differentiated according to their objective value (a higher objective value gets higher weight).
3. *Resampling:* Resampling takes place to transport the particles from the low-weight points to the high-weight ones. A new particle set is formed with the same number of particles ($N_{s}$). The new particles (${\overset{˜}{x}}_{k}^{i}$) are randomly drawn from the old ones, each with the probability corresponding to its weight. Thereby, the high-weight old particles are replaced by equally weighted new ones, while the low-weight ones disappear. The probability distribution of the particles remains the same during the resampling, only the weights are equalized again. The sample impoverishment caused by this step is compensated by the particle move step of the next iteration to maintain particle diversity.

More information and a detailed analysis about the transition noise $u_{k}^{i}$ and the distribution type and steepness of $\varphi_{k}(\cdot )$, in addition to a thorough description of the resampling strategy can be found in a previous work in [24]. The pseudocode of the PFO concept for global optimization is summarized in Algorithm 1.

**Algorithm 1:**
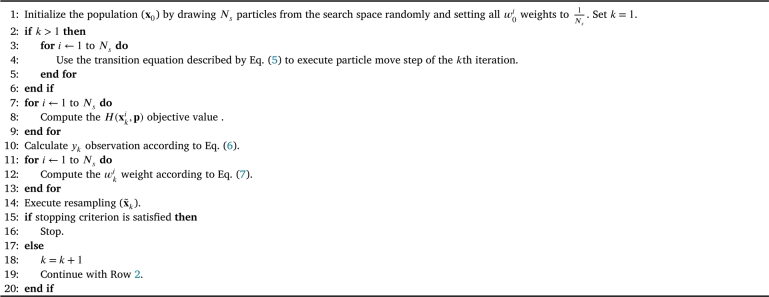
Pseudocode of the general PFO algorithm.

Algorithm 1 summarizes the general PFO framework for finding the global optimum, which is actively investigated. Different goal-oriented improvements have been provided in the recent years, e.g., to increase global optimization performance [33], to perform multi-objective optimization [34] or to apply it for dynamic path planning [35]. Its performance was verified by tests on many benchmark functions [33]. The authors of this paper also suggested some improvements together with a comparative analysis on the different versions, and a clustering-based supplement of the algorithm to analyze the propagation of the particles and their accumulation around optimums [24]. However, the applicability of PFO has not yet been investigated for robust optimization. Therefore, the following sections provide a detailed concept for improving the algorithm in this direction by integrating uncertainty handling into it. As the PFO concept is based on probabilistic principles, it has a quite promising potential for optimization under uncertainty problems where multiple uncertain factors have to be handled.

#### 2.2.2 Robustness aspects in a process system

Robustness of a solution, algorithm, or operating point of a technology refers to its stability, i.e., the ability of keeping high performance even under uncertainties outside and inside the system. The environment of the systems is often volatile, and there are inherent random phenomena in the system as well. Moreover, our knowledge about the system is always incomplete, and the created models never describe the reality entirely. Solutions based on these imprecise information may not reach the theoretically expected performance in practice.

During optimization, engineers search for the “best” solution to a problem, e.g., the “best” structure of a designed equipment, or the “best” operating point of a technology. The term “best” refers to the optimization goal; however, it can be considered from two aspects. On the one hand, performance of the optimum is important. On the other hand, its stability can be examined keeping in mind that our knowledge about the technology and the future is uncertain, and some phenomena are unpredictable. Uncertainties are often neglected, and only the first aspect is considered. In this case, the global optimum of the objective function is enough to search for. However, when the optimal solution is transplanted into reality, it may yield much lower performance than expected. In these cases, in the presence of multiple uncertain factors, whose effect is significant on the objective function, the stability is also worth considering besides the performance during the search.

**Figure 2:**
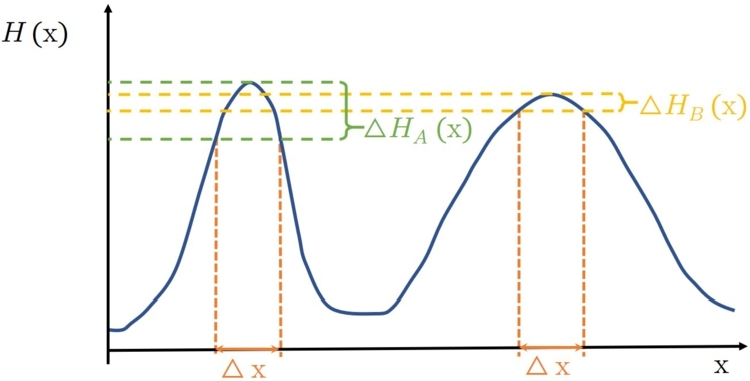
Illustration of optimum robustness.

To investigate the term *robust optimum* in depth, an illustrative example stands here, supported by Fig. 2.

In the schematic example of Fig. 2, a one-dimensional objective function with two peaks is considered. One of them is somewhat higher (and also sharper) than the other, this is the global optimum here, while the other has lower peak value with less sharpness, this a local optimum. From two optimums, one is more robust than the other, if the same uncertainty level of *x* causes less variation on the objective value. In this case, $\Delta H_{A}(x)>\Delta H_{B}(x)$, therefore, the local optimum with a lower peak value is more robust.

Robustness can be considered regarding the decision variables, on the one hand. In this case, just as the example before, the objective function has multiple peaks, which should be compared based on the requirements and user preferences on performance and robustness as two conflicting factors to choose the preferable peak. On the other hand, robustness can be defined regarding the objective function (containing system model) parameters as well. In this case, there may not be multiple peaks of the objective function, but its shape may change non-linearly by the effect of the uncertain parameters. Thereby, the optimal solution to reach the highest expected performance may not be identical with the optimum besides the nominal values of the parameters.

These two aspects of robustness have to be handled in a different way during optimization. Therefore, the remaining part of Section 2.2 that introduces the improvements of the PFO algorithm for robust optimization is divided following this logic, and Section 3 also contains the experiment results accordingly.

#### 2.2.3 Integrating decision variable robustness into PFO

**Figure 3:**
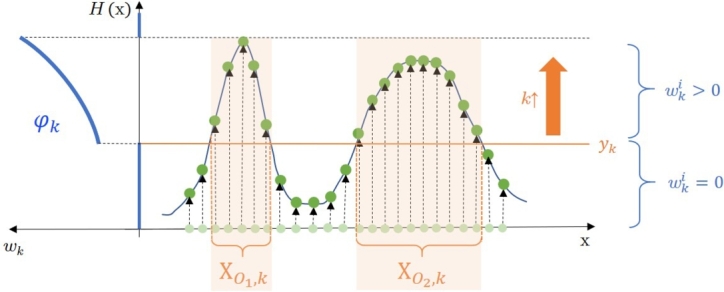
Illustration of the presence of peak robustness aspect besides peak height in the general PFO algorithm. $X_{O_{1},k}$ and $X_{O_{2},k}$ denotes the set of the positive-weight particles at the *k*th iteration (besides *y*_*k*_) in case of the *O*_1_ and *O*_2_ optimums, respectively. *φ*_*k*_ is also illustrated, which defines the *w*_*k*_ weights of the particles according to their *H*(*x*) objective values.

One of the big advantages of the PFO optimization scheme is that it takes into account the robustness of the peaks besides their objective value by default, resulting from its structure. To take a look at this issue in depth, let us consider a schematic example introduced in Fig. 3, similar to the one in Fig. 2.

Looking at Fig. 3, it is clear that in case of equidistantly distributed particles on the search space (which is the initial situation in the search), more particle will get positive weights around a flatter peak, i.e., more robust optimum, than around a sharper one under the same $y_{k}$ level. Regarding Fig. 3, set $X_{O_{2},k}$ contains more elements than the set $X_{O_{1},k}$. Thereby, more particles will propagate to the next iterations in case of the less sharp peak (assuming that the difference between the objective values on the top of the peaks is not too high).

Of course, the objective values also affect the propagation and accumulation of the particles. If the sharper peak is much higher, the neighborhood of the other will be impoverished in particles as the continuously elevated $y_{k}$ level reaches the top of it, assuming that the search has not been ended before. On the other hand, the tunable steepness of the φ(⋅) function also controls the balance between performance (peak height) and robustness (peak width) aspects by giving a preference to particles with higher objective value, which means that the particles on the top of the higher peak will get higher weights than the ones on the top of the other, as can also be seen in Fig. 3.

It is clear from the above-mentioned example, that the consideration of the opposing aspects of optimum performance and robustness can be controlled by the steepness of the φ(⋅) exponential density function. It can be expressed as:

$$\varphi_{k}(v_{k})=\left\{ \begin{array}{c} \frac{\lambda_{k}e^{\lambda_{k}v_{k}}}{\int_{0}^{H_{max,k}-H_{min,k}}\lambda_{k}e^{\lambda_{k}v_{k}}},\;\text{if}\;0\leq v_{k}\leq H_{max,k}-H_{min,k}\\ 0,\;\text{otherwise} \end{array}\right.$$

where

(8) $$\lambda_{k}=\frac{\log\Delta w}{H_{max,k}-H_{min,k}}$$

with the adjustable parameter Δ*w* representing the weight ratio of the best and worst particle in the current particle set if all the particles would be kept ($y_{k}=H_{min,k}$), i.e., it refers to the steepness of the function. If Δw=1, φ(⋅) becomes a uniform distribution, i.e., all the particles above the $y_{k}$ objective value level will get equal weights. In fact, this Δ*w* parameter makes the φ(⋅) function dynamically changing during the search, prioritizing the particles with higher objective values as described in Appendix A.

Increasing the value of Δ*w* results the increased emphasis on performance during the search, at the expense of robustness. If its value is extremely high, the consideration of robustness aspect is negligible. As for the opposite direction, if a small Δ*w* value is used, the robustness also matters besides the performance. However, Δ*w* cannot be decreased below 1, as in this case, the particles with lower performance would get higher weights, which would mislead the search. Thereby, this minimum value defines a border regarding the consideration of robustness at the expense of performance. If shifting the balance more significantly towards robustness is desired, another idea is necessary.

In this paper, the idea of adding an extra, latent perturbation on the particle positions is suggested to solve this problem. This means that the position of the particle to be propagated is still $x_{k}^{i}$ as calculated by Equation (5), but its objective value is evaluated at a different position in its neighborhood (${\overset{ˆ}{x}}_{k}^{i}$) for Equation (7), which can be determined as:

(9) $${\overset{ˆ}{x}}_{k}^{i}=x_{k}^{i}+{\overset{ˆ}{u}}_{k}^{i}={\overset{˜}{x}}_{k-1}^{i}+u_{k}^{i}+{\overset{ˆ}{u}}_{k}^{i}$$

where ${\overset{ˆ}{u}}_{k}^{i}$ denotes the latent perturbation noise on $x_{k}^{i}$, which is sampled from the uniform distribution on the [−Δx,Δx] interval in this paper. However, it has to be noted that if the user has a guess on the distribution of the uncertain realization of **x**, than it can also be used instead, regardless of its type.

Thereby, ${\overset{ˆ}{u}}_{k}^{i}$ noise can be a factor in setting the balance between performance and robustness aspects during the search, together with the φ(⋅) density function. The higher Δ*x* is, the larger neighborhood of $x_{k}^{i}$ considered during the search, and the more robustness aspect is taken into account. During the resampling step, multiple particles arrive at the same point. If the $u_{k}^{i}$ perturbation noise that moves the particles ensuring the exploration of the search space is already not too large, then particles remain around the resampled points and ${\overset{ˆ}{u}}_{k}^{i}$ ensures the exploration of the neighborhood of them.

**Figure 4:**
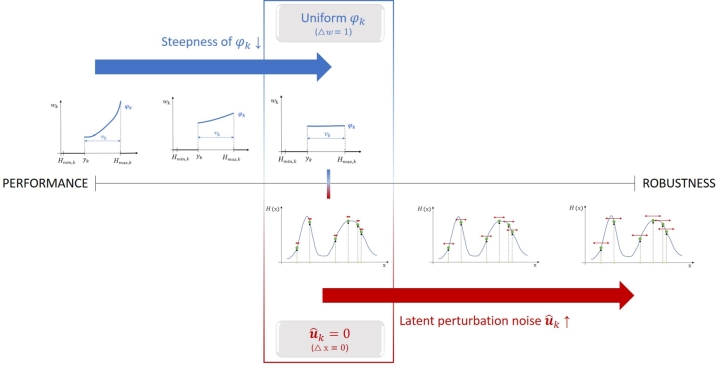
The proposed tuning method regarding the performance-robustness scale.

The suggested tuning method described above is summarized in Fig. 4: starting from the edge belonging to the performance, robustness aspect can be emphasized by decreasing the steepness of φ(⋅) until it becomes a uniform distribution, then for further amplification of robustness aspect, ${\overset{ˆ}{u}}_{k}^{i}$ latent perturbation noise is introduced, and its variance is increased by the Δ*x* parameter.

Taking a look at Fig. 4, the question arises of which point of the scale the switch point belongs to. The switch point is the point where $\varphi_{k}$ is uniform (Δw=1) and ${\overset{ˆ}{u}}_{k}=0$ (Δx=0). Therefore, the question is how much the PFO algorithm takes into account performance and robustness under these parameter settings. It relates to the question that under a given parameter setting (that defines a point of the performance-robustness scale) which optimum of the objective function will be the most preferable during the search, i.e., which one the particles will accumulate in. In terms of these issues, a theoretical explanation is given below.

Consider two optimums with ID $O_{1}$ and $O_{2}$ of an objective function. (For simplicity, a one-dimensional objective function is used for illustration.) The question is which factors influence the search and how, where the particles are forced to move. The dominance of one of the peaks over the other at the *k*th iteration depends on the relation between the two terms of the inequality described by Equation (10). (At first, ${\overset{ˆ}{u}}_{k}=0$ is assumed.)

(10) $$\int_{X_{O_{1},k}}\varphi_{k}(H(x))dx\overset{?}{⋚}\int_{X_{O_{2},k}}\varphi_{k}(H(x))dx$$

Equation (10) investigates the relation between the integral of the weights regarding the two optimums that need to be compared. The same $\varphi_{k}(\cdot )$ function is used (which depends on $y_{k}$ as seen in Equation (7)), however, different intervals of the search space is considered ($X_{O_{1},k}$ and $X_{O_{2},k}$, where the weights are positive). These intervals also depend on $y_{k}$, and where it cuts the function while it is elevated (Fig. 3). If the term on the left side of Equation (10) is greater than that on the right, the $O_{1}$ optimum dominates in iteration *k*, and particles start moving towards its neighborhood. Thereby, the proportion of particles around this optimum increases during this iteration.

If $\varphi_{k}$ is defined as a uniform distribution, then the positive-weight particles are not weighted according to their objective value, i.e., their weights are equal, and independent from their objective value. In this case Equation (10) simplifies to the following form:

$$d(X_{O_{1},k})\overset{?}{⋚}d(X_{O_{2},k})$$

where the domination of the peaks only depends on the length (d(⋅)) of the $X_{O_{1},k}$ and $X_{O_{2},k}$ interval which is defined by the current $y_{k}$. In this case, the objective values affect only indirectly the search while the objective function is scanned over while $y_{k}$ level is elevating.

If $\varphi_{k}$ is defined as an exponential distribution, the original form defined by Equation (10) needs to be used. Generally, the proportion of the particles around the optimums ($W_{O_{n},k}$) can be determined recursively as:

$$W_{O_{n},k}=\frac{W_{O_{n},k-1}\int_{X_{O_{n},k}}\varphi_{k}(H(x))dx}{\sum_{i=1}^{N_{O}}W_{O_{i},k-1}\int_{X_{O_{i},k}}\varphi_{k}(H(x))dx}$$

where $N_{O}$ represents the number of optimums. If the integrals equal to each other, i.e., none of the optimum dominates the other(s), distribution of the particles between the optimums remains the same. If the $W_{O_{i},k-1}$ proportions are equal to each other (which is the case, e.g., at the first iteration), the distribution after iteration *k* will correspond to the $\int_{X_{O_{i},k}}$ integral values. As the $y_{k}$ level elevates during the search, these integral values change, and their cumulated effect appears on the current distribution of the particle set.

The last important matter regarding these theoretical investigations is the effect of the latent perturbation noise (${\overset{ˆ}{u}}_{k}$), which value was considered zero above. However, if this perturbation noise is used for tuning or for representing physical uncertainty on the decision variables (Δx≠0), its effect can be integrated to the equations above by estimating the integrals in Equation (10) using the expected value of the objective value at *x* on the [x−Δx,x+Δx] interval instead of its nominal value:

$$\int_{X_{O_{n},k}}\varphi_{k}(H(x))dx\to \int_{X_{O_{n},k}}\varphi_{k}(E_{\left[x-\Delta x,x+\Delta x\right]}(H(x)))dx$$

where $E_{\left[\cdot ,\cdot \right]}(\cdot )$ denotes the expected value on a given interval.

**Figure 5:**
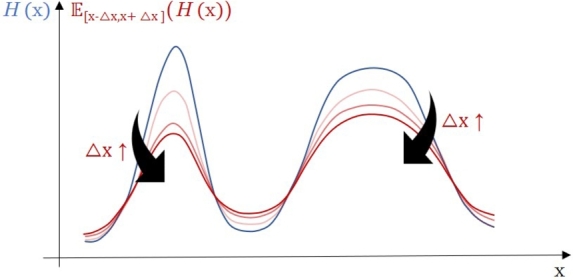
Changes of the objective function if instead of each point, the expected value of them in a fixed neighborhood ([*x* − Δ*x*,*x* + Δ*x*]) is considered. As Δ*x* is increased, the peaks are shifted more and more.

The higher Δ*x* is set, the wider neighborhood of the nominal value of *x* is considered during the search. Thereby, in this case, a modified objective function is optimized theoretically, as seen in a schematic illustration in Fig. 5.

Fig. 5 shows schematically how the original objective function changes if the expected value of a neighborhood of *x* is considered instead of the current point of the search space. The height of the peaks and may even their position are shifted. Thereby, the more robust peak becomes higher after a while.

Of course, the above-mentioned is only correct in this form, if the search space and uncertainty space are sampled perfectly (infinite number of particles). However, during the PFO search, these integrals and terms are approximated by a finite sample. Nevertheless, the equations above describe analytically the processes that take place behind this Monte Carlo (MC) method. By using an MC-based algorithm, the complete sampling of the search and uncertainty spaces can be avoided, thus the computational demand can be decreased, besides making the optimization goals easily achievable.

The tuning method proposed in this section using the steepness of $\varphi_{k}$ and a latent perturbation noise to consider performance and robustness aspects will be verified by a sensitivity analysis on an objective function with multiple optimum with different optimum performance and robustness in Section 3.1. It will be introduced, how the search can be controlled through the tuning of the algorithm according to the preferences of the users. The updated version of the algorithm regarding this issue (PFO-X algorithm) is summarized in Algorithm 2.

**Algorithm 2:**
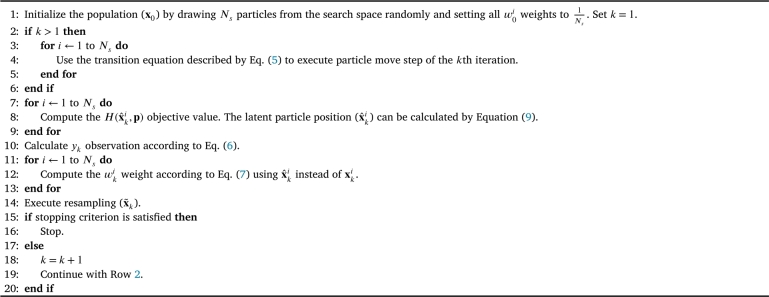
Improved version of the PFO algorithm for robust search under decision variable uncertainty (PFO-X algorithm).

#### 2.2.4 Integrating parameter robustness into PFO

Besides the probabilistic nature of the decision variables, parameters of the objective function including the system model can also be uncertain. The system model parameters are usually fitted to some noisy measurement data; thereby, their estimated values can be given only together with a confidence interval. The model structure is also imprecise usually, because it is a simplified description of reality built on assumptions, and some phenomena are even hidden from our eyes which could not be described. On the other hand, the objective function that defines the optimization aim can have uncertain parameters as well, e.g., because of the unpredictability. These parameters define the shape of the objective function, which can be different under their different values.

Parameters can influence the altitude of the peaks of the objective function representing the optimums, and also the position of them. If this effect is nonlinear (assuming that the parameter uncertainty can be modeled by a symmetric PDF with mean equal to the nominal value) then best solution on the search space differs from the peak position under the nominal parameter values. Thereby, this robust solution gives a better expected performance if the examined parameter is uncertain.

To explore this phenomenon and integrate this robustness aspect as an additional search criteria into the algorithm, the particle move step of the general algorithm described by Algorithm 1 was extended. In this case, besides perturbing the position of the particles on the search space (Eq. (5)), the uncertain parameters are also sampled from their distribution for each particle in each iteration as:

(11) $$p_{k}^{i}∼D(p_{nominal}),$$

where $p_{k}^{i}$ marks the vector contains uncertain parameters belonging to the *i*th particle in the *k*th iteration, sampled from $D(p_{nominal})$ containing the set of distributions of uncertain parameters. D(⋅) is defined according to our a priori knowledge about the uncertain nature of the parameters, with the expected value equal to the nominal values of the parameters ($p_{nominal}$). Its shape can be any type (e.g., uniform or normal) depending on the nature of the parameter and source of uncertainty, or even an empirical distribution can be used.

To gain the PFO-P algorithm introduced in Algorithm 3, only two modifications were made to the general PFO algorithm described by Algorithm 1. Firstly, Step 5 was introduced, which enables to take into account the uncertainty of the parameters. Secondly, the objective function values of the particles are not calculated from the fix values of the parameters in Step 9 any more, but the new sampled parameter values ($p_{k}^{i}$) are used in case of each particle.

**Algorithm 3:**
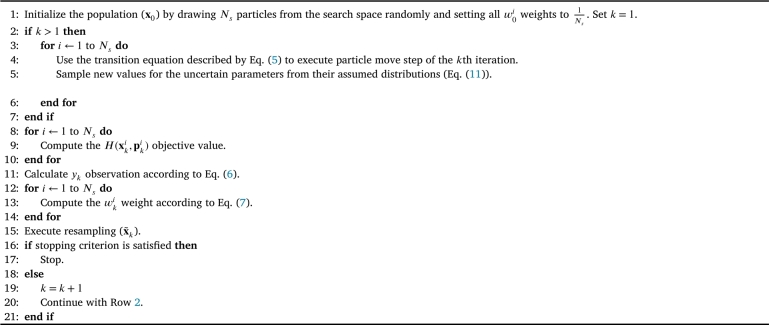
Improved version of the PFO algorithm for robust search under parameter uncertainty (PFO-P algorithm).

It will be seen in Section 3.2, that these two simple modifications that can be easily integrated into the general algorithm are enough to find robust optimum regarding parameter uncertainty. Moreover, if necessary, the uncertainty of multiple parameters can be handled and considered simultaneously without any prior calculations, if their probability distributions are available.

#### 2.2.5 General considerations on the PFO algorithm for robust optimization

As can be seen in Section 2.2.3-2.2.4, both the parameter and decision variable uncertainties are handled by adding an extra perturbation on the particles. Thereby, the variance of their objective values will also be larger, and some elements of the algorithm have to be adapted to this changed condition.

Originally, the $v_{k}$ random number of Equation (6) is sampled from the $\varphi_{k}(\cdot )$ probability density bounded by the lowest ($H_{min,k}$) and highest ($H_{max,k}$) objective values of the current particle set. However, if the perturbation that ensures robust optimum search is added to the parameters and / or decision variables, the $\left[H_{min,k},H_{max,k}\right]$ interval becomes much wider and its width fluctuates more along the iterations. Thereby, choosing a random number ($v_{k}$) from the $\left[H_{min,k},H_{max,k}\right]$ interval in each iteration to define the $y_{k}$ level (Eq. (6)) that specifies the border of zero-weight and positive-weight particles may mislead the search by keeping only a few particles (with parameter values that cause extremely high objective values) for the next iteration and neglecting all the others.

Therefore, in case of robust optimum search, a gradually elevating $y_{k}$ level that is fixed to the current particle set is more suitable such as:

$$v_{k}=\alpha (H_{max,k}-H_{min,k})$$

where *α* defines a percentage where to cut the $\left[H_{min,k},H_{max,k}\right]$ interval. In our experiments, α=0.3 was used which was found an ideal value keeping enough but not too many particles. Thereby, the elevation of $y_{k}$ and the weighting of the particles, that were both depending on the $\varphi_{k}(\cdot )$ density before, can be handled independently.

As another issue, the $u_{k}$ trajectory also has to be updated in case of robust optimization. The most preferable trajectory of the standard deviation of $u_{k}$ along the iteration is an exponentially decreasing one as was shown in [24]. At large value of *k*, it approximates zero, but never reaches it. However, in case of robust optimization where the particles are perturbed, this property makes the search very long. After the particles are concentrated around some points of the search space and properly cover their neighborhood, it is worth setting $u_{k}$ to zero for the rest of the iterations. Thereby, the algorithm regroups the particles amongst these fixed points that cover the neighborhood of potential optimums and accumulate them around the best region.

The computational tractability is also a key issue in case of sampling-based methods, that can be represented by analyzing the computational complexity of the technique. Here (because the number of iterations is not constant but depends on the fulfillment of the stopping criteria) the computational complexity of a single iteration is examined, similarly to the previous analysis of the general PFO [24].

The computational complexity of the PFO algorithm can be given depending on the dimension of the optimization problem ($N_{d}$), the uncertain decision variables ($N_{d,x}$) and the uncertain parameters ($N_{d,p}$), and the number of particles ($N_{s}$). For the PFO-X algorithm, it can be formalized as:

$$O(PFO\text{-}X)=O(TR)+O(FE)+O(OC)+O(WC)+O(RE)==O(N_{S}\times N_{d})+O(Cof\times N_{S}\times N_{d,x})+O(Coo)\;+O(N_{S}\times Cow)+O(N_{S}\times Cor)$$

where TR, FE, OC, WC and RE denote the transition, function evaluation, observation calculation, weight calculation and resampling steps, respectively. *Cof*, *Coo*, *Cow* and *Cor* represent the cost of function evaluation, observation calculation, weight calculation and resampling, respectively. The creation of the latent particle position appears in the function evaluation steps, that complexity thus depends on the dimension of uncertain decision variables ($N_{d,x}\leq N_{d}$).

Analogously, the computational complexity of the proposed PFO-P algorithm can be defined as:

$$O(PFO\text{-}X)=O(TR)+O(FE)+O(OC)+O(WC)+O(RE)==O(N_{S}\times N_{d}\times N_{d,p})+O(Cof\times N_{S})+O(Coo)\;+O(N_{S}\times Cow)+O(N_{S}\times Cor)$$

where the dimension of uncertain parameters ($N_{d,p}$) have an importance in the transition step.

The number of function evaluations in a run can be defined as:

$$N_{function\;evaluations}=N_{iterations}N_{s}$$

where mainly the number of iterations ($N_{iterations}$) is affected by the dimension of uncertain factors ($N_{d,x}$ and $N_{d,p}$) as the time needed for the convergence of the algorithm increases by the number of uncertain factors and the degree of uncertainty. (On the other hand, as the degree of uncertainty of the problem increases, more and more particles are needed to gain unequivocal results. However, the analysis and definition of sufficient particle number for a given robust optimization problem is not the topic of this paper, as creating an appropriate tuning strategy regarding $N_{s}$ needs further investigations.)

## 3 Case studies on the robust optimization PFO frameworks

The ideas introduced in Section 2.2 to improve the PFO algorithm and make it applicable for robust optimization were tested on various optimization problems. Regarding decision variable robustness, Algorithm 2 and the proposed tuning technique were tested on an appropriate benchmark function, and the results are shown in Section 3.1. On the other hand, parameter robustness issues are examined on a practical case study of a styrene reactor in Section 3.2.

### 3.1 Searching for robust optimum regarding the decision variables

In this section, the robustness regarding the decision variables is investigated. In case of a multi-modal objective function, it is an interesting question which peak is the most preferable (optimal) if robustness aspects are also considered besides the performance. The general PFO algorithm and its improved version (PFO-X algorithm) are tested for these kinds of problems here, and the results are analyzed based on the concepts in Section 2.2.3.

#### 3.1.1 Finding the most robust global optimum

The first experiments were executed on the Branin-function, which has multiple global optimums with the same performance but different robustness. This two-dimensional function takes the following form, considering a maximization task:

$$H(x)={\left(x_{2}-\frac{5.1}{4\pi^{2}}x_{1}^{2}+\frac{5}{\pi }x_{1}-6\right)}^{2}+10(1-\frac{1}{8\pi })\cos x_{1}+10$$

**Figure 6:**
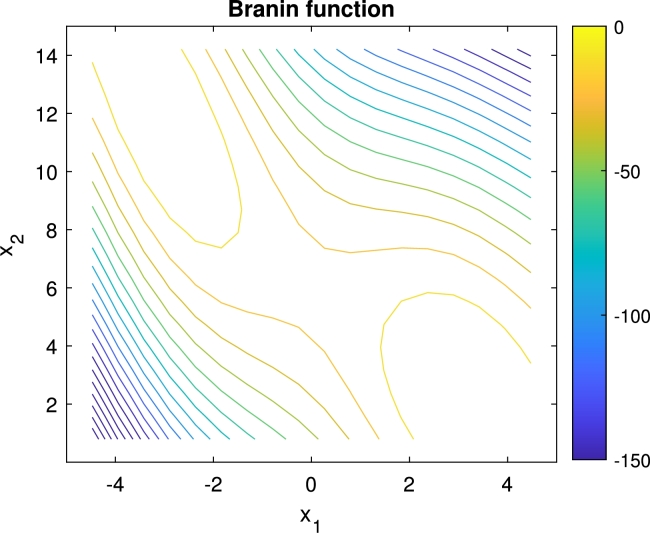
Branin-function on the space $x_{1}\in \left[-5,5\right]$, $x_{2}\in \left[0,15\right]$.

The Branin function was examined on the space $x_{1}\in \left[-5,5\right]$, $x_{2}\in \left[0,15\right]$. The function has two global optimums on this space at $x^{⁎}=(\pi ,2.275)$ and (−π,12.275) with the objective value $H(x^{⁎})=0.397887$. The (π,2.275) optimum is more robust than the other, as declared in [36]. The contour plot of the function is shown in Fig. 6.

**Figure 7:**
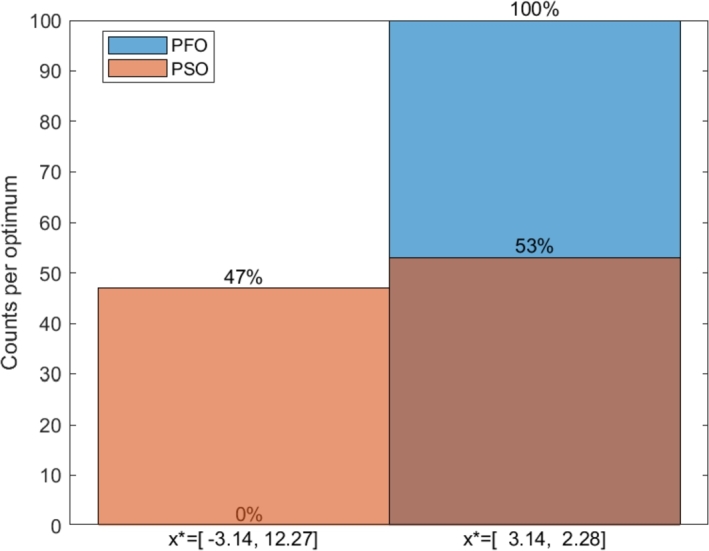
Comparison of the optimization results of the PFO and PSO algorithms on the Branin function. 100 runs were executed in both case, and *N*_*s*_ = 100 particles used.

The optimization of the Branin function was tested using the general PFO algorithm (Algorithm 2) compared to the Particle Swarm Optimization (PSO) algorithm. The results of 100 runs using $N_{s}=100$ particles in both cases are summarized in Fig. 7.

It can be seen in Fig. 7, that even the general PFO algorithm (without any improvements regarding robustness) performs quite well. The PFO algorithm found the more robust global optimum all the time, even in case of a uniform $\varphi_{k}$, in contrast to the PSO algorithm which found the two global optimums about half and half. This phenomenon can be explained by the integrals in Equation (10). As was declared in Section 2.2.3, the PFO algorithm takes some account of robustness by default. Since in this case two equally high peak were considered, no one peak is favored based on the performance, and the differentiation according to the robustness aspect can dominate during the search.

In conclusion for this part, if the user has to determine the most robust one from multiple equally high peaks of the objective function, the general PFO algorithm is suggested to be used for this aim, as it finds efficiently and robustly this peak without any improvements of it.

#### 3.1.2 Handling performance-robustness trade-off

If the optimums of the objective function differ from each other in performance and robustness as well, the search becomes more complex, and the developed PFO-X algorithm (Algorithm 2) is suggested to be used in this case. The optimal solution for each user depends on their preferences, which can be defined by choosing a point from the performance-robustness scale in Fig. 4. For the different intervals of the scale, different peaks form the optimal robust solution. Choosing a point from the scale representing the preferences of the users can be performed by setting the Δ*w* and Δ*x* parameters of the algorithm, that refer to the steepness of the $\varphi_{k}$ probability density describing the differentiation based on performance and the level of latent perturbation noise (${\overset{ˆ}{u}}_{k}$) strengthening the domination of robustness aspect.

To investigate and verify the proposed tuning method in Section 2.2.3, an appropriate benchmark function with multiple local optimums with different robustness and performance was created. The benchmark function was created by the summarization of multiple one-dimensional Gaussian function as:

$$H(x)=\sum_{i=1}^{10}\kappa_{i}e^{-\frac{{\left(x-\mu_{i}\right)}^{2}}{2\sigma_{i}^{2}}}$$

where the vectors μ=[−14;−10.5;−7;−3;2;4;12;21;26;32], σ=[1.8;1.8;1.8;0.5;1;1;3;2;1;1] and κ=[1.1;1.1;1.1;1.7;1;1;1.5;1.55;1.6;1.2]. *μ* defines the location of the peaks, *σ* the width of them and *κ* the height of them. Flat peaks were formed by adding multiple peaks near each other.

**Figure 8:**
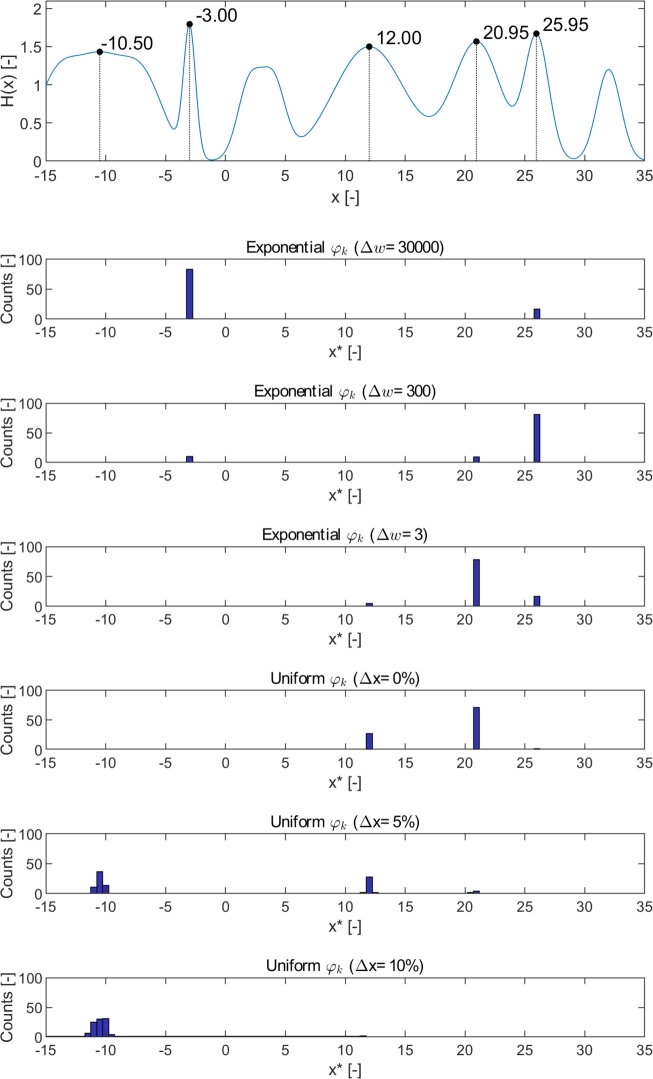
Sensitivity analysis on the created Gaussian-based test function along the performance-robustness scale. The plot on the top illustrates the objective function with seven local maximums, and the subplots below show the results (found optimums) of 100 runs of the PFO-X algorithm on histograms. The titles of the subplots include the values of the tuning parameters that were used, based on the concept in Fig. 4. The labels of the peaks refer to the optimum positions.

The created objective function together with the optimization test results is shown in Fig. 8. The PFO-X algorithm was used for these experiments besides different parameter sets along the performance-robustness scale. The tuning parameters were set by starting from a high value of Δ*w*, reducing it to one while Δ*x* is keeping zero, and then increasing Δ*x* while keeping Δ*w* at one which is its minimal value. A defined parameter set, i.e., a point of the performance scale defines a trade-off level between performance and robustness aspects. Scrolling through the scale, it was expected that different peaks would be found as the optimal solution, related to the current trade-off level.

Fig. 8 verifies the idea that the proposed tuning scale using Δ*w* and Δ*x* as tuning parameters is suitable to represent the performance-robustness scale. It can be seen, that in case of an extremely large value of the Δ*w* (left edge of the scale belonging to the performance aspect), the global optimum (at x=−3.00) is found in almost all the cases. The larger the value of Δ*w* is, the more the performance of the peaks dominates and the more efficient two peaks with similar height (e.g., at x=−3.00 and x=25.95 here) can be distinguished. On the other hand, as we move towards the robustness aspect of the scale in Fig. 4 (from top to bottom in the subplots) the peaks with second, thirds, etc. largest peaks (with increasing robustness) are found optimal. It can be also seen, that the peak at x=3.38 and x=32.01 are never found optimal, as they neither possess a better performance nor are more robust than, e.g., the peak at x=−10.50. Another observation is that the centers of the sharp peaks are found precisely; however, the found optimums in case of the flat peaks (e.g., x=−10.50, x=12.00) have a wider distribution, because of the nearly constant objective function on the top of the peak.

**Figure 9:**
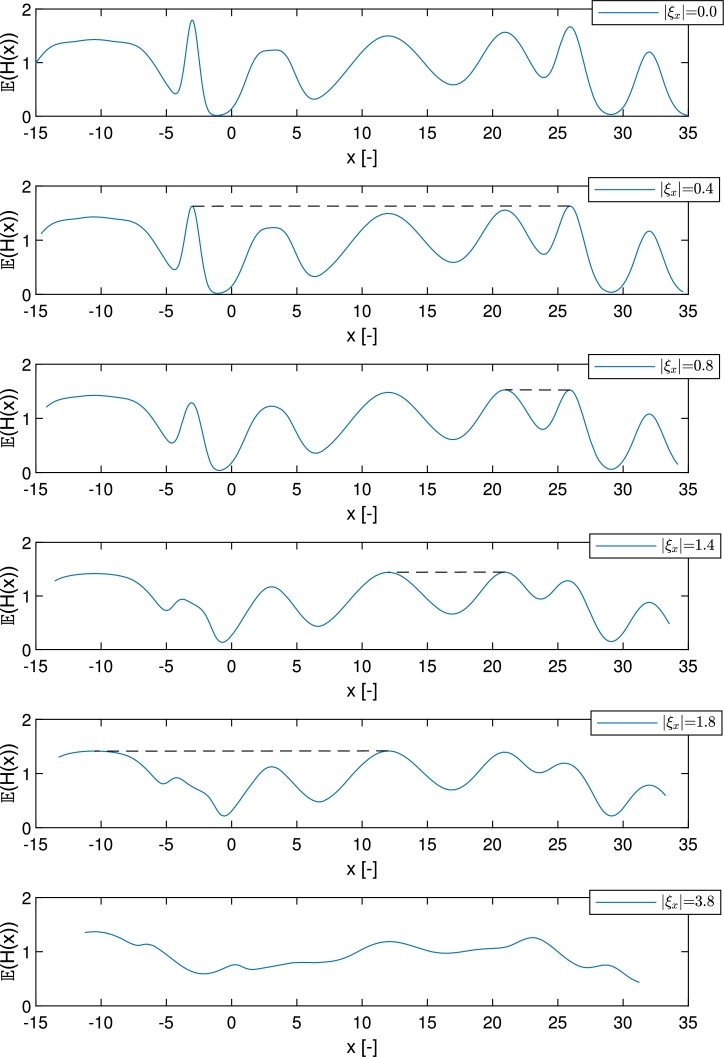
Expected objective value curves under different uncertainty levels of *x* (|*ξ*_*x*_|). Dashed lines connect the optimums with equal height in each case.

The expected value curve of the objective function that is introduced in Fig. 9 besides different $\xi_{x}$ uncertainty levels also corresponds to these results. It can be seen, that as uncertainty on *x* increases, the global optimum becomes lower and after a while, the peak with the second, third, etc. largest performance becomes the highest as the shape of the expected value curve (which forms the latent objective function in case of PFO-X algorithm) is changing. In Fig. 9, the values of $\xi_{x}$ where the optimal solution changes its positions, i.e., where the two highest peaks of the current expected value curve become equally high (marked by dashed lines) are shown. Thereby, for example, if $0.8<|\xi_{x}|<1.4$, the peak at x=20.95 is the highest, i.e., it is the robust optimal solution in case of this uncertainty level of *x*.

The question arises that which value of $\xi_{x}$ a defined trade-off level represented by (Δw,Δx) corresponds to, i.e., which form of the latent expected value curve is optimized under a given (Δw,Δx) parameter set. To investigate this issue, the two scales need to be brought to a common ground.

**Figure 10:**
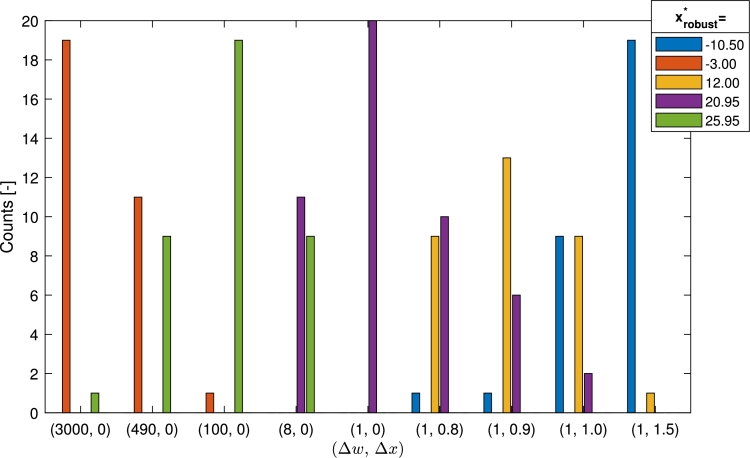
Grouped bar chart of the found robust optimums under different (Δ*w*,Δ*x*) parameter sets. In each case, the results of twenty independent runs are introduced. Colors represent the five optimums with different robustness as can be seen in the legend.

The boundary points on the scale between the robust optimums, i.e., where the robust optimal solution changes its position, in case of the $\xi_{x}$ scale can be determined based on the results of Fig. 9. The $\xi_{x}$ values where the two highest peaks take equal performance at the top are searched for, and it can be seen that these are 0.4,0.8,1.4 and 1.8. This means, that when $|\xi_{x}|<0.4$ for example, the robust optimum is at x=3.00, but if $0.4<|\xi_{x}|<0.8$ it is at x=25.95. On the other hand, on the (Δw,Δx) scale, a sensitivity analysis was executed to find the points belonging to the above-mentioned $\xi_{x}$ values, i.e., the parameter sets where two peaks are found as a robust optimum with equal probability. During the tuning, $N_{s}=3000$ particles were used, as it was found that using more does not change the results significantly, however, does increase the computational demand. The results regarding the found optimums in 20 runs, corresponding to the boundary points (at (Δw,Δx)={(490,0),(8,0),(1,0.8),(1,1.0)}) and parameter sets between them (where it is expected to find unequivocally one optimum) are shown in Fig. 10. The gained boundary points are consistent with the results in Fig. 8.

As can be seen in Fig. 10, two-two optimums were found with nearly equal probability at the boundary points, and one optimum dominates the others between them. This means, that at the boundary points, the robust performance of the two peaks are nearly equal, and the user can choose any of them without losses on robust performance. Between the boundary points, one of the optimums dominates significantly, i.e., that optimum is found in all or almost all the runs. The only exception is $x_{robust}^{⁎}=12.00$; at (Δw,Δx)=(1,0.9), the optimum at x=20.95 also appears a few times. The reason for this is the extremely close performance values they have as can be seen in Fig. 9 looking at $|\xi_{x}|=1.4$ and 1.8.

**Figure 11:**
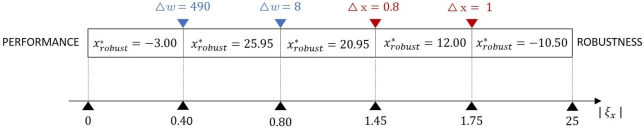
Robust optimal solutions on the performance-robustness scale together with the determined boundaries regarding *ξ*_*x*_ decision variable uncertainty and the (Δ*w*,Δ*x*) tuning parameters (corresponding to Fig. 4) of the PFO-X algorithm. The edges of |*ξ*_*x*_| were defined as zero and the half of the length of the search space ($\frac{UB-LB}{2}$).

The established performance-robustness scale based on the results, showing the bounds between the optimums regarding $\xi_{x}$ decision variable uncertainty and the (Δw,Δx) is illustrated in Fig. 11.

The scale in Fig. 11 summarizes the boundary points between the robust optimal solutions and gives a guide to the user on how to set the tuning parameters to find a robust optimum for a certain $\xi_{x}$ uncertainty level. Thereby, the (Δw,Δx) tuning scale was fit to the uncertainty level scale. However, this fitting seems to be objective function specific, and has to be set again in case of another objective function, which is a major disadvantage of the methodology at the moment, and set out the subject of a future research, namely, developing a general scaling technique.

**Figure 12:**
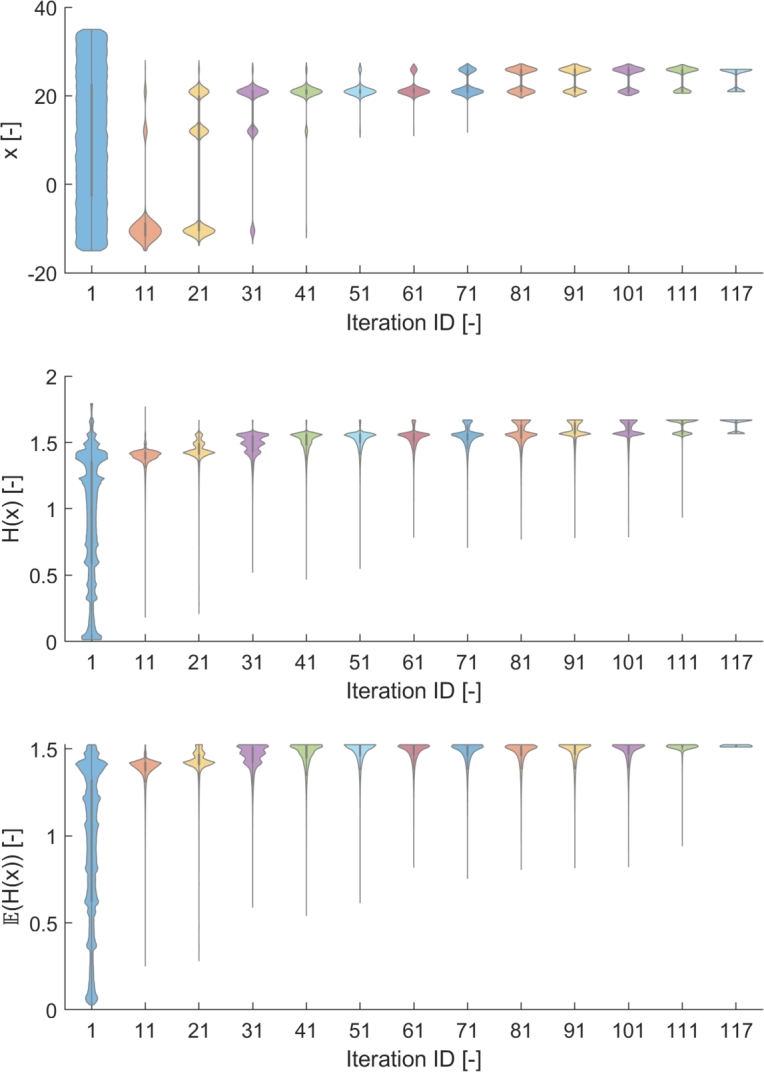
Propagation of particles during the search with (Δw,Δx)=(8,0) parameters, that belongs to the |*ξ*_*x*_| = 0.8 uncertainty level. The position (*x*) and the objective value (*H*(*x*)) of the particles are represented by violinplots. These data were collected during the optimization. The mean objective value (E(H(x))), however, is calculated for each particle, considering the corresponding uncertainty range that is [*x* − |*ξ*_*x*_|,*x* + |*ξ*_*x*_|]. This is a summary of 20 runs.

The correctness of the fitted scale was validated by investigating the distribution of particles regarding the belonging decision value, objective value, and mean objective value in the related uncertainty range ($\xi_{x}$) along the iterations. An example of these results can be seen in Fig. 12, which belongs to the (Δw,Δx)=(8,0) parameter set of the PFO algorithm, and the $|\xi_{x}|=0.8$ uncertainty level on the other hand. In this case, our expectation is to find the optimums at x=25.95 and x=20.95 with equal probability, based on the previous results. The *x* and H(x) values were obtained by collecting the data of particles along the iterations during the optimization with PFO-X algorithm with parameters (8,0), and the E(H(x)) values were gained by computing the mean objective value on the $\left[x-|\xi_{x}|,x+|\xi_{x}|\right]$ interval for all particles. The related empirical probability distributions are illustrated by violinplots, and the aggregated results of 20 runs are shown in Fig. 12.

The aggregated results of 20 runs in Fig. 12 verify that particles are found in both expected optimums at the end of the search. Looking at the objective values it is clear that they have slightly different objective values, however, the mean objective values at the two optimums under the current uncertainty level are the same, as only one peak can be seen on the plot of E(H(x)) at the end. This fact verifies that at the $|\xi_{x}|=0.8$ uncertainty level these two optimums have equally good performance (as was also seen in Fig. 9).

### 3.2 Robust optimization under parameter uncertainty

The proposed method for handling parameter uncertainty in Section 2.2.4 was tested on the optimization problem of an adiabatic plug flow styrene reactor, whose detailed model together with its validation results are provided in Appendix B. The optimization of the chosen styrene reactor example was investigated many times, e.g. in [37] and [38]. However, neither of the works paid attention to the uncertainties which are important factors from practical aspects. In this work, the profit-optimal operating conditions of the reactor as described in Appendix B.3 are searched for, under parameter uncertainty assumed on the activation energy ($E_{i}$) of the main reaction. A ±10% uncertainty is considered, modeled by a uniform distribution according to the estimation accuracy of this kind of parameters based on measurement data [39]. This parameter affects the shape of the objective function significantly as will be introduced later.

The profit function contains the molar flowrates of the components in the product besides their prices (Equation (B.6)). These flowrates are computed by the reactor model from the input variables like input temperature, steam flowrate, pressure, and ratio of raw materials. From these, the input pressure ($P_{0}$) and the ratio of the two input flows (ethylbenzene and superheated steam) marked by *SOR* were chosen as the decision variable. Thereby, a two-dimensional search space was obtained.

**Figure 13:**
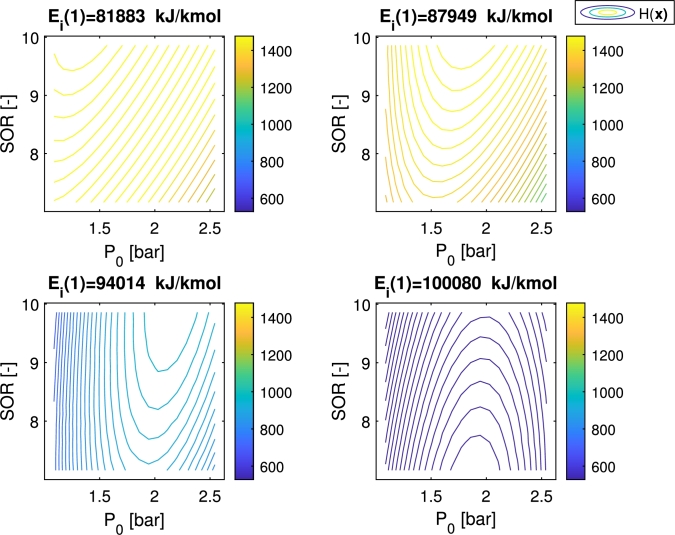
Effect of a ±10% on the activation energy of the main reaction (*E*_*i*_(1)) on the objective function (*H*(*x*)). The objective function is illustrated by contour plots on the two-dimensional search space under four different values of *E*_*i*_(1) from the uncertainty range. The nominal value of *E*_*i*_(1) is 90981 kJ/kmol as shown in Table B.1.

The profit of the technology (which is the output of the objective function) depends nonlinearly on the activation energy of the main reaction ($E_{i}(1)$) through the reactor model. As can be seen in Fig. 13, the objective function has one optimum and changes its position in the constrained search space under the different values of $E_{i}(1)$ and an uncertainty range of ±10%.

**Figure 14:**
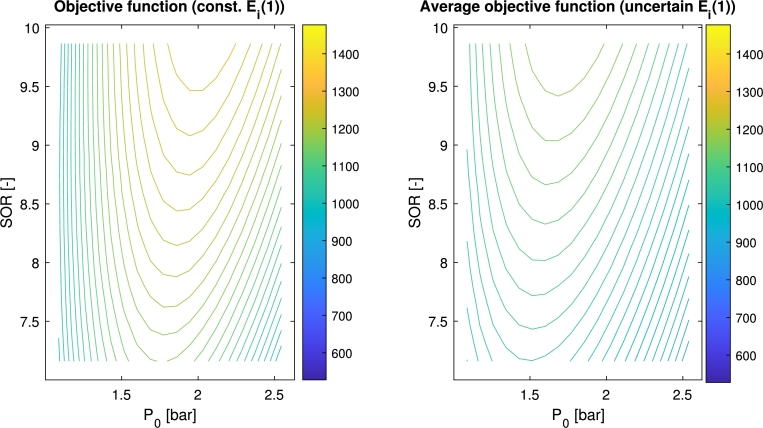
Comparison of the objective function assuming *E*_*i*_(1) takes its constant nominal value (on the left) and the average objective function curve besides 10% uncertainty on it (on the right).

The average curve of the objective function under this ±10% uncertainty range on $E_{i}(1)$ considering uniform distribution is compared with the objective function in case of the nominal value of $E_{i}(1)$ in Fig. 14.

It is clear from Fig. 14, that the original objective function takes its optimum at a different position than the averaged curve that represents the expected position of the parameter robust optimum as well. Executing the optimization, using the PFO-P algorithm in the robust optimization case, the numerical results summarized in Table 1 also verify this statement and are consistent with Fig. 14.

**Table 1:** The found global and robust optimum using the PFO-P algorithm. The table contains the mean and standard deviation of the decision variables in 20 runs with *N*_*s*_=250 particles.

Decision variable	Global optimum	Robust optimum
*P*_0_	2.0323 ± 0.0105	1.7811 ± 0.1080
*SOR*	10.0096 ± 0.0151	9.7798 ± 0.1512

**Figure 15:**
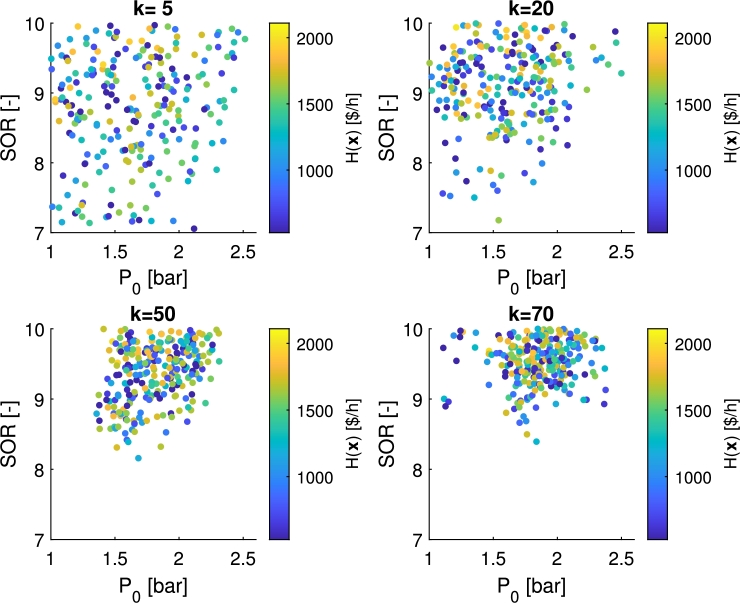
Propagation of the particles in the robust search with PFO-P with 10% parameter uncertainty on *E*_*i*_(1). The position of the 250 particles can be seen on the 2D search space, and their objective values represented by their color according to a fixed colorbar.

The propagation of the particles together with their objective values during one of the robust search runs are illustrated in Fig. 15.

It can be followed by Fig. 15, how the particles are concentrating in the preferable region and how they are concentrated more and more around the robust optimum during the iterations. Their current objective values are represented by their colors. As can be seen, the neighborhood of the robust optimum also contains blue, i.e., low performance points, because of the randomly sampled value of the uncertain parameter in every iteration for each particle. However, it is also seen, that there are proportionally more points with high objective values (yellow ones) in the neighborhood of the optimum than further from it.

To verify the fact that the operating point represented by the found robust optimum performs better than that belonging to the global one, the objective values of them were compared under different values of the $E_{i}(1)$ parameter. (It was sampled corresponding to the probability distribution was used during the optimization to represent it.)

**Figure 16:**
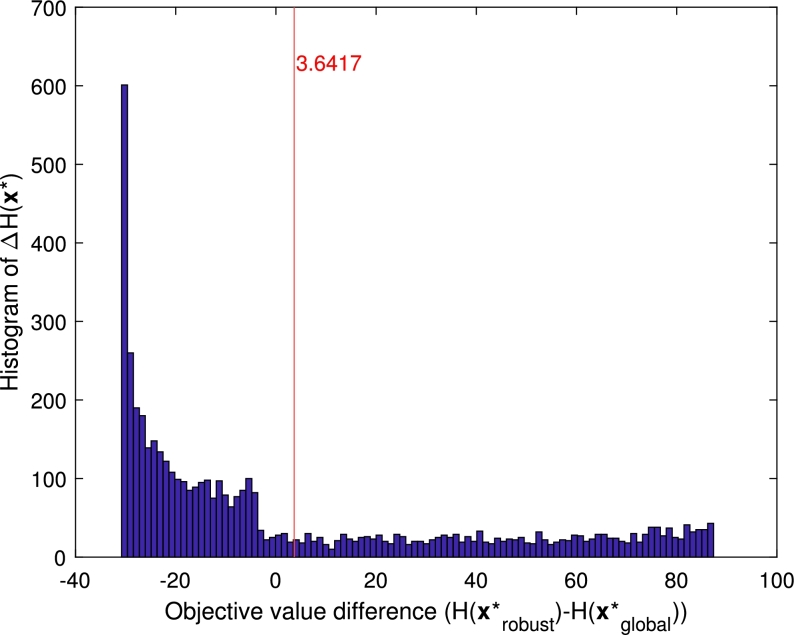
Objective value difference between the robust ($H({x^{⁎}}_{robust}$) and the global optimum ($H({x^{⁎}}_{global}$) under 5000 different values of *E*_*i*_(1) sampled from its assumed uncertainty range. The mean of the results is shown by a vertical red line.

Fig. 16 shows the histogram of the objective value difference between the robust and global optimum in case of different values of the uncertain parameter. It can be seen, that the expected objective value difference marked by red line is positive, thereby, the robust optimum is expected to perform better than the global optimum if we have only this kind of uncertain knowledge about $E_{i}(1)$.

## 4 Conclusion

This work verified the applicability of the PFO algorithm for robust optimization aims. In a technological system, many kinds of uncertainties can appear, which have to be handled when performing an optimization task. We proposed a probabilistic methodology to overcome this problem, providing improvements on the PFO algorithm by integrating sampling-based uncertainty handling into it, regarding parameter and decision variable robustness, as well.

The proposed method was tested on benchmark functions and also on a practical case study. In case of decision variable robustness, it was seen that the PFO algorithm always finds the most robust optimum of an objective function with multiple global optimums with equal peak values. From this aspect it was shown that the PFO definitely outperformed the PSO algorithm. Furthermore, a technique for handling performance-robustness trade-off was also provided based on the proposed PFO-X algorithm. It was shown that all the optimums with different sharpness and performance can be found, if the user systematically changes the tuning parameters which define the corresponding decision variable robustness. A case study on fitting the tuning scale to the uncertainty scale was also provided on a one-dimensional benchmark function with multiple local optimums.

The performance of the proposed method for parameter robustness was explored on a case study of a styrene reactor as a practical example. It was seen, that parameter uncertainty affects the shape of the objective function significantly. The improved version of the PFO algorithm (PFO-P) was able to find the robust optimum. Operating the technology at this operating point, the expected revenue is greater than in the case of the optimum found by fixing the parameter at its nominal value.

**Main advantages:** Based on the results, the PFO algorithm seems quite promising in the field of robust optimization. The proposed improvements make it possible to find robust optimums under parameter and decision variable uncertainties as well. Besides, one of the big advantages of the suggested sampling-based strategy is that it can be used for uncertainties with any kind of probability density. It was also seen, that without any improvements, the general PFO itself always finds the most robust one from multiple global optimums.

**Limitations:** The use of the improved PFO considering decision variable robustness needs further investigations. The introduced case study verified, that with proper parameterization, all the optimums belonging to the different points of the performance-robustness scale can be found. However, the determined parameter sets related to certain values of the physical realization of uncertainty may not be valid in the case of another objective function. Otherwise, the computational demand increases with the number of decision variables, just like in the case of any other population-based optimization technique. Here, the number of uncertain factors and their variance affect the required computational effort.

**Future directions:** One topic of our future research is clarifying the connection between the physical realization of the uncertainty and the tuning parameter scale if possible. Besides, the dependence of the required number of particles on the number of uncertain parameters, their variance, and the complexity of the problem is planned to be clarified, and a tuning strategy given for this parameter as well. Moreover, we would like to examine the possibility of integrating multiple and/or different types of uncertainties during the search.

The proposed method could be relevant in engineering problems where uncertainty needs to be considered during the optimization, e.g., in case of a production planning problem, where the prices are volatile in time which aims to find the optimal operational trajectory belonging to maximal profit, thus facilitating decision-making from an inventory perspective. As prices vary over time, and only forecasts that always have an error can be used, the objective function is dynamic, and its parameters are uncertain, on the one hand. On the other hand, as a technological system is examined, whose variables are non-deterministic in practice, process and input uncertainties can also be considered, and model uncertainties need to be taken into account. The proposed method can be suitable for any similar optimization problems for stationery or dynamic systems, even if the assumed distribution of some uncertain factors also changes in time (e.g., an uncertain parameter representing catalyst activity may decrease over time, or uncertainty of prices grows as we look further and further ahead).

## Ethics statement

This article does not contain any studies involving human participants.

## CRediT authorship contribution statement

**Éva Kenyeres:** Writing – original draft, Software, Methodology, Conceptualization. **Alex Kummer:** Writing – review & editing, Supervision, Conceptualization. **János Abonyi:** Supervision, Conceptualization.

## Declaration of Competing Interest

The authors declare that they have no known competing financial interests or personal relationships that could have appeared to influence the work reported in this paper.

## Figures and Tables

**Figure A.1:**
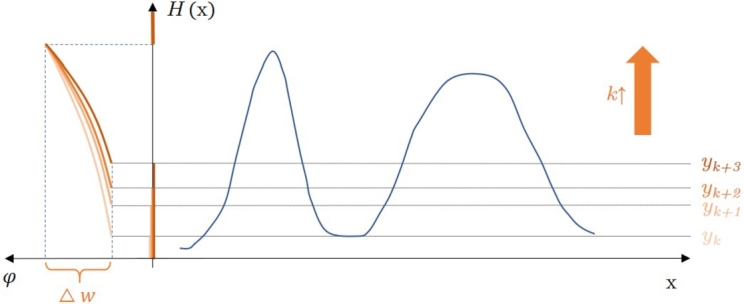
Illustration of the changing nature of the unnormalized *φ*(⋅) weighting function during the search. The elevation of the *y*_*k*_ objective value level (defining the lower boundary of positive weight particles) forces the population to an increasingly narrow objective value range. Thereby, the steepness of the weighting function fitted to the best and worst particles of the current population becomes higher, thus differentiating the particles in a higher resolution.

**Figure B.2:**
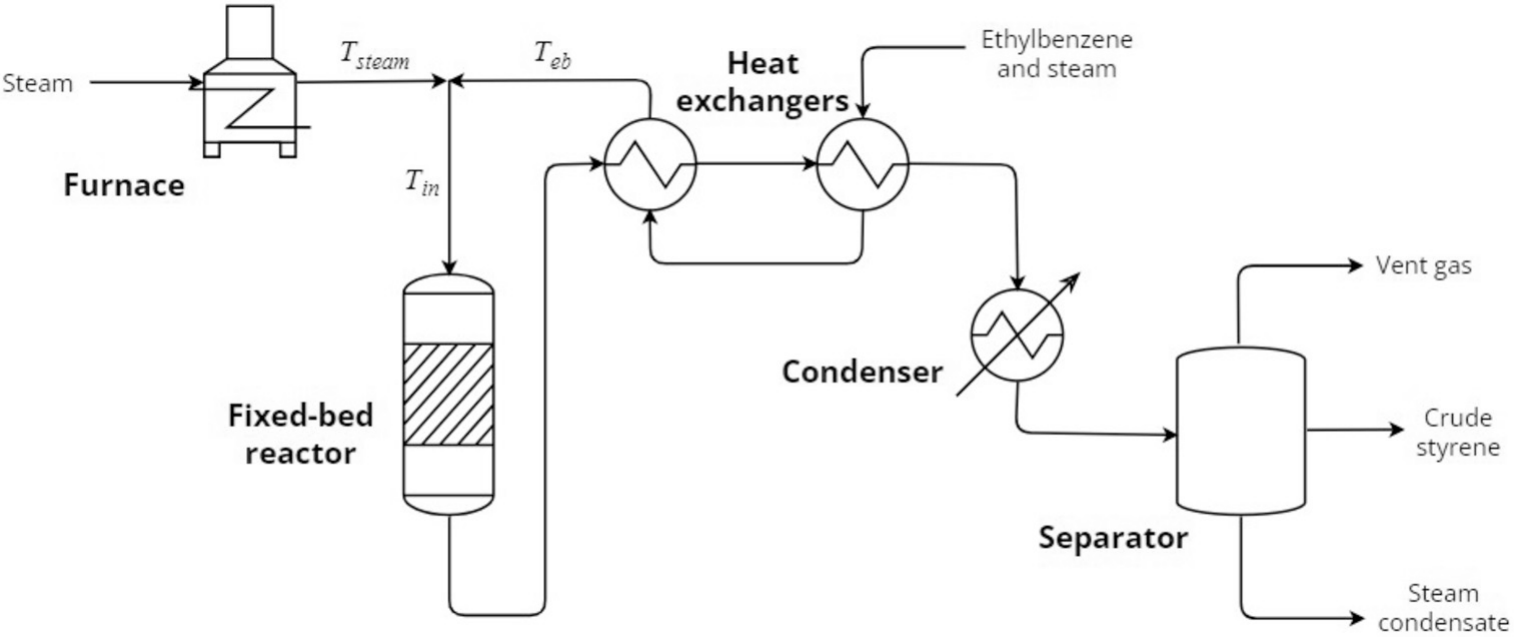
Process flow diagram of styrene production technology [41]. *T*_*steam*_ and *T*_*eb*_ refer to the temperature of the two feed streams which are mixed before the reactor inlet. *T*_*in*_ denotes the temperature of the mixture.

**Table B.1:** The reactions and their rate expressions with rate constants. The rate coefficients are calculated by Arrhenius-equation as *k*_*i*_ [kmol kg^−1^ s^−1^ bar${}^{-n}$] = exp$\left(A_{i}-\frac{E_{i}}{RT}\right)$; and the partial pressures from the total pressure according to the molar ratios as $p_{j}=\frac{F_{j}}{\sum_{j=1}^{N_{c}}F_{j}}P$. *K*_*eb*_ marks the equilibrium constant of the main reaction, which is temperature dependent as *K*_*eb*_ [bar] = exp$\left(-\frac{122725-126.3T-0.002194T^{2}}{8.314T}\right)$[41].

Reaction ID	Reaction	Rate expression	*A*_*i*_ [-]	*E*_*i*_ [kJ/kmol]
1		$r_{1}=k_{1}(p_{eb}-p_{st}p_{H_{2}}/K_{eb})$	-0.0854	90981.4
2		*r*_2_ = *k*_2_*p*_*eb*_	13.2392	207989.2
3		$r_{3}=k_{3}p_{eb}p_{H_{2}}$	0.2961	91515.3
4		$r_{4}=k_{4}p_{steam}p_{eth}^{0.5}$	-0.0724	103996.7
5		*r*_5_ = *k*_5_*p*_*steam*_*p*_*meth*_	-2.9344	65723.3
6		*r*_6_ = *k*_6_*p*_*steam*_*p*_*CO*_*P*/*T*^3^	21.2402	73628.4

**Table B.2:** Validation of the system model with the available literature data.

Data source	Industrial data (1964-1966) [42]	Sheel's model results (1969) [42]	Elnashaie's model results (1994) [44]	Yee's model results (2003) [41]	Chaudhari's model results (2022) [38]	Our model (2023)
*F*_*eb*_ [kmol/h]	19.45	19.02	19.39	19.63	19.48	**19.64**
*F*_*st*_ [kmol/h]	15.57	15.56	15.57	15.40	15.33	**15.37**
*F*_*be*_ [kmol/h]	1.50	1.50	1.48	1.44	1.49	**1.46**
*F*_*to*_ [kmol/h]	2.03	2.03	2.08	2.05	2.06	**2.06**
*F*_*eth*_ [kmol/h]		0.34	0.33			**0.32**
*F*_*meth*_ [kmol/h]		0.74	0.53			**0.52**
*F*_*steam*_ [kmol/h]		448.12	447.62			**447.72**
$F_{H_{2}}$ [kmol/h]		21.65	22.62			**22.27**
*F*_*CO*_ [kmol/h]		0.03	0.03			**0.03**
$F_{CO_{2}}$ [kmol/h]		2.48	2.73			**2.68**

*T* [K]	850.00	850.76	851.98	849.75	852.93	**850.25**
*P* [bar]	2.32	2.32	2.36	2.33	2.32	**2.32**

**Figure B.3:**
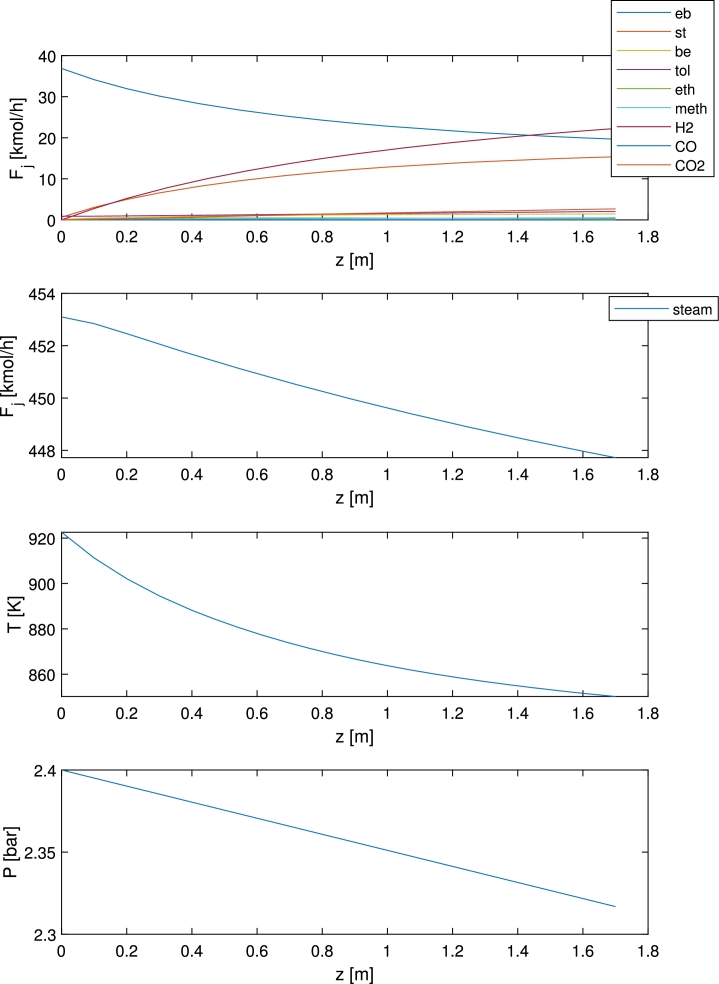
Molar flow rates, temperature and pressure profiles along the reactor length.

**Table B.3:** Prices used in Equation (B.6) for product and raw materials. The cost of raw materials is shown in a negative form.

Component ID	Prices [$/kmol]
*G*_*st*_	106.57
*G*_*to*_	-109.32
*G*_*be*_	-81.78
$G_{H_{2}}$	1.37
*G*_*meth*_	4.43
*G*_*eth*_	7.17
*G*_*steam*,1_	-0.0495
*G*_*steam*,2_	-0.0003

**Table C.1:** Constant values for calculating the heats of reactions by Equation (C.1). The values are taken from [44], except the *a*_1_ value which was misstated there so it was taken from [42] converting it to SI unit.

Reaction ID	*a*_*i*_ [kJ/kmol]	*b*_*i*_ [kJ/kmol/K]
1	120750.11	4.56
2	108818.11	-7.96
3	-53178.20	-13.19
4	82065.74	8.84
5	211255.19	16.58
6	-45223.66	10.47

**Table C.2:** Values of constants appearing in Equation (C.2) and (C.3) required for calculating the specific heat of the components [46].

Organic components	*α*_*j*_ [kJ/kmol/K]	*β*_*j*_ [kJ/kmol/K^2^]	−10^5^*γ*_*j*_ [kJ/kmol/K^3^]
ethylbenzene (eb)	9.34	0.460	15.36
styrene (st)	17.04	0.417	13.85
benzene (be)	-1.71	0.325	11.06
toluene (to)	2.41	0.391	13.07
ethylene (eth)	11.84	0.120	3.65
methane (meth)	14.15	0.075	1.80

Inorganic components	*a*_*j*_ [kJ/kmol/K]	10^3^*b*_*j*_ [kJ/kmol/K^2^]	10^−5^*c*_*j*_ [kJ⋅K/kmol]

steam (H_2_O)	28.85	12.06	1.01
hydrogen (H_2_)	27.01	3.51	0.69
carbon monoxide (CO)	28.07	4.63	-0.26
carbon dioxide (CO_2_)	45.37	8.69	-9.62

**Table C.3:** Parameters of the model equations [41], [42], [44].

Symbol	Name	Value
*ρ*_*b*_	catalyst bulk density [kg/m^3^]	2146
*d*	reactor diameter [m]	1.95
*L*	reactor length [m]	1.70
*G*_0_	total mass velocity [kg/m^2^/s]	1.138
*ε*	bed void fraction [-]	0.445
*D*_*p*_	catalyst particle diameter [m]	0.0047
*μ*_*G*_	viscosity of the mixture [Pa⋅s]	3⋅10^−5^
*R*	gas constant [J/mol/K]	8.314
$F_{steam}^{eb}$	amount of steam introduced with the ethylbenzene [kmol/h]	50.4
*N*_*c*_	number of components [-]	10
*N*_*r*_	number of reactions [-]	6

**Table C.4:** Operating conditions of the industrial reactor [41]. In the ethylbenzene feed, styrene, toluene and benzene impurities are present, as well.

Quantity	Value
Ethylbenzene in the feed ($F_{eb}^{0}$ [kmol/h])	36.87 (95.7%)
Styrene in the feed ($F_{st}^{0}$ [kmol/h])	0.67 (1.7%)
Benzene in the feed ($F_{be}^{0}$ [kmol/h])	0.11 (0.3%)
Toluene in the feed ($F_{to}^{0}$ [kmol/h])	0.88 (2.3%)
Steam feed ($F_{steam}^{0}=F_{eb}^{0}\text{SOR}$ [kmol/h])	453.1
Inlet temperature (*T*_*in*_ [K])	922.59
Inlet pressure (*P*_*in*_ [bar])	2.4

## Data Availability

Data will be made available on request. For requesting data, please write to the corresponding author.

## Appendix A Dynamically changing nature of the weighting function

The weighting function φ(⋅) of the PFO algorithm defines the differentiation between the positive weight particles in every iteration. Its steepness is tuned by the Δ*w* parameter, that represents the weight ratio of the particles with highest and lowest performance in the current population. Because of this definition of Δ*w*, the φ(⋅) function has a dynamically changing nature during the iterations, so that the particles with very high performance (near to the optimums) are prioritized more and more.

To understand the changing nature of the φ(⋅) function, Fig. A.1 illustrates a representative example. The $y_{k}$ value is monotonically increasing during the search, causing that the particles concentrate in smaller and smaller objective value intervals.

As the φ(⋅) function is fitted to the “best” and “worst” particle in the current population (their weight ratio is fixed), it changes during the search. The objective value of the “worst” particle increases monotonically, together with the $y_{k}$ limit. Thereby, the positive weight particles are differentiated by higher and higher resolution as the absolute steepness of the φ(⋅) rises (while its relative steepness regarding the current population represented by Δ*w* remains the same).

## Appendix B Case study of a styrene reactor

The features of the styrene reactor and the validation of its model are introduced in comparison to the results available in the literature. Then, the optimization problem of the reactor is defined.

### B.1 Introduction of the styrene reactor model

As the earlier papers did not contain all the necessary information in one place, and sometimes printing mistakes even appeared, this section aims to provide a carefully collected and proper information set about the model, which can help others who want to reproduce the simulation in the future, such as the validation of the model and the profit function to be optimized.

#### B.1.1 Technological background

Styrene is one of the most important monomer, which is used to produce polystyrene or different copolymers e.g. acrylonitrile-butadiene-styrene (ABS), styrene-acrylonitrile (SAN) or styrene-butadiene rubber (SBR). These plastics are widely used in construction (e.g. pipes, tanks), packaging (e.g. foams) and automotive (e.g. tyres, reinforced plastics, automobile body putty) because of their advantageous properties [40].

Styrene is made by the dehydrogenation of ethylbenzene. Since this reaction is endothermic and reversible, high temperature is preferable, however, it also promotes the formation of by-products e.g. benzene and toluene. The reactions take place in a fixed-bed catalytic reactor to increase conversion and selectivity. The ethylbenzene feed is diluted by superheated steam in order to achieve the required feed temperature, to decrease the partial pressure of ethylbenzene and hydrogen, thus forcing the equilibrium reaction to the direction of product formation and to prevent the formation of coke at the catalyst surface. The process flow diagram of the technology can be seen in Fig. B.2.

Many papers have studied the modeling of the styrene reactor technology. One of the first was published in 1969 by Sheel and Crowe, which used industrial data from the Polymer Corporation, Sarnia, Ontario, thus fitting the kinetic coefficients of the model [42]. This study formed the basis for several subsequent works (e.g. [43], [44], [45]).

In this paper, the pseudo-homogeneous adiabatic model presented by Yee *et al.* was chosen as a basis which was shown to be more suitable for optimization aims than the heterogeneous one [41]. It is important to note that this pseudo-homogeneous model does not deal with the diffusion of the chemical species in the pores of the catalyst, thus the fitted reaction rates using this model are suitable only for the specific reactor beyond the same catalyst properties where the industrial data was gained from [44]. The only modification made in the model of Yee *et al.* is that the molar balance was used instead of conversion similarly to [38].

The reactor was assumed plug flow with radial uniformity and adiabatic, which means that the whole amount of necessary superheated steam was introduced at the inlet of the unit. The catalyst activity was considered constant lack of available data similarly to [41]. The mass and heat transfer limitations and the diffusion in the catalyst pellet are contained by the rate constants. Thereby, the steady-state model of the reactor can be described by the following equations:

(B.1)

(B.2)

(B.3)

where $F_{j}$, *T* and *P* are considered in kmol/s, K and bar, respectively.

Equation (B.1) represents the molar balance. In the considered 6 reactions (shown in Table B.1 with their rate expressions and rate constants) a total of 10 components are included, which, together with their notations in the brackets used later in this article to refer to them, are as follows: ethylbenzene (eb) and steam (steam) as feed, styrene (st) as the main and toluene (to) and benzene (be) as side products which are saleable, at the same time ethylene (eth), methane (meth) and some inorganic species as carbon monoxide (CO), carbon dioxide (CO_2_) and hydrogen (H_2_) are also formed. Their formation or loss are expressed by $R_{j}$ where *j* refers to the component:

(B.4) $$R_{j}=\sum_{i=1}^{N_{r}}\nu_{ij}r_{i}$$

where $\nu_{ij}$ represents the stoichiometric coefficient of the *j*th component in the *i*th reaction (which is negative in case of reactants).

Equation (B.2) defines the heat balance. The temperature along the reactor length is only affected by the heats of reactions as the unit is considered adiabatic. The temperature dependence of the heat of reactions ($\Delta H_{i}$) and specific heat ($c_{p,j}$) was taken into account. The details of this issue can be found in Appendix C. The inlet temperature ($T_{in}$), which forms the boundary condition of Equation (B.2), can be calculated by energy balance from the two feed streams, considering some steam ($F_{steam}^{eb}$) is introduced with the ethylbenzene at $T_{eb}$ according to industrial practice to prevent undesirable reactions before the reactor [41]:

(B.5) $$T_{in}=\frac{F_{eb}^{0}c_{p,eb}T_{eb}+(F_{eb}^{0}\text{SOR}-F_{steam}^{eb})\;c_{p,steam}T_{steam}+F_{steam}^{eb}c_{p,steam}^{eb}T_{eb}}{F_{eb}^{0}c_{p,eb}+(F_{eb}^{0}\text{SOR}-F_{steam}^{eb})\;c_{p,steam}+F_{steam}^{eb}c_{p,steam}^{eb}}$$

where *SOR* refers to the steam over reactant (ethylbenzene) molar ratio which includes the amount introduced with the ethylbenzene as well, $F_{eb}^{0}$ to the ethylbenzene feed molar flow rate, and $c_{p,steam}^{eb}$ to the specific heat of the steam but in the temperature of ethylbenzene feed ($T_{eb}$).

Equation (B.3) presents the pressure drop along the fixed bed by Ergun equation, where $\rho_{G}$ represents the density of the gas mixture which is calculated by the ideal gas law as explained in Appendix C. The viscosity of the gas mixture ($\mu_{G}$) is considered constant according to [42].

All the other notations representing parameters and variables that appear in the equations above are defined by their names and values, as well as the inlet conditions of the reactor in Appendix C.

### B.2 System analysis of the styrene reactor model

The system model was solved using ODE23s solver in MATLAB. The calculation sequence was similar to the one presented in [38]. Although Chaudhari *et al.* used ODE15s, ODE23s was found more suitable for us because of numerical mistakes which caused negative molar flow rates.

The results were compared to the former ones found in the literature shown in Table B.2.

As seen in Table B.2, our results are close to the industrial data (the relative error is 0-3%) and similar to that of the former modelers (e.g. comparing to the results of Elnashaie, the relative error is less than 2% in every case). The molar flow rate, temperature and pressure profiles along the reactor length can be seen in Fig. B.3.

### B.3 Optimization problem of the styrene reactor

The optimization task of the styrene reactor was defined to search for the profit-optimal operating conditions regarding two decision variables: the inlet pressure ($P_{0}$) and the steam over reactant ratio (*SOR*). The converted form of the profit function in the work of Sheel *et al.* was used, which includes the prices, and the molar flow rates the inlet steam temperature as [42]:

(B.6) $$Profit=G_{st}F_{st}+G_{to}F_{to}+G_{be}F_{be}+G_{H_{2}}F_{H_{2}}+G_{meth}F_{meth}+G_{eth}F_{eth}+(G_{steam,1}+G_{steam,2}T_{steam}^{0})F_{steam}$$

where the molar flowrates ($F_{j}$) are given in kmol/h, and the prices ($G_{j}$) in $/kmol. The inflated prices (for 2024) are summarized in Table B.3.

The flowrates in the profit function are calculated by the system model using the decision variables as inputs. Thereby, the objective function includes the system model and the profit function, as well. The decision variables are bounded as [41]:

(B.7) $$1\leq P_{0}\leq 2.63\;\text{bar}$$

(B.8) 7≤SOR≤20,

and a technological constraint is also considered, regarding the inlet temperature of the feed mixture ($T_{in}$):

(B.9) $$850\leq T_{in}\leq 925\;\text{K}.$$

## Appendix C Parameters and operating conditions of the styrene reactor

In this section, all the data is collected which is necessary to solve the styrene reactor model introduced in Section B.1.

First of all, the heat balance (Equation (B.2)) requires the heats of reactions ($\Delta H_{i}$) and specific heat values ($c_{p,j}$) of the components. Their temperature dependencies are expressed the following way. In case of the heats of reactions:

(C.1) $$\Delta H_{i}=a_{i}+b_{i}T$$

the specific heat for organic components:

(C.2) $$c_{p,j}=\alpha_{j}+\beta_{j}T+\gamma_{j}T^{2}$$

and for inorganic ones:

(C.3) $$c_{p,j}=a_{j}+b_{j}T+\frac{c_{j}}{T^{2}}$$

where subscript *i* refers to the *i*th reaction and *j* to the *j*th component similarly to Appendix B. The required constant values are presented in Table C.1, Table C.2.

In the Ergun equation (Equation (B.3)), the $\rho_{G}$ was calculated according to the ideal gas law as:

(C.4) $$\rho_{G}=\frac{P\bar{M}}{RT}$$

where the total pressure (*P*) should be given in kPa and $\bar{M}$ represents the average molar mass of the mixture which was computed according to the molar ratios:

(C.5) $$\bar{M}=\sum_{j=1}^{N_{c}}M_{j}\frac{F_{j}}{\sum_{j_{1}}^{N_{c}}F_{j}}$$

The cross-sectional area ($A_{t}$) of the reactor is calculated from its diameter (*d*).

The meaning of other notations used in Equation (B.1)-(B.3) and their values are collected in Table C.3.

The operating conditions of the industrial system that serve as the boundary conditions of Equation (B.1)-(B.3) are presented in Table C.4. Although the molar flow rates are given in kmol/h here, it has to be noted that during the computation they have to be handled in kmol/s because of the dimension of $k_{i}$. The composition of the catalyst was used is 62% Fe_2_O_3_, 36% K_2_CO_3_, 2% Cr_2_O_3_.

## Footnotes

1 Note: In case of the PFO algorithm, the problem always has to be defined as a maximization task.
